# Supplementary material for: Evaluation and Projection of Surface PM2.5 and Its Exposure on Population in Asia Based on the CMIP6 GCMs
Source: Int J Environ Res Public Health. 2022 Sep 24;19(19):12092. doi: 10.3390/ijerph191912092 (PMC9566559; doi:10.3390/ijerph191912092)
Supplement: Supplementary file 1 [file ijerph-19-12092-s001.zip › ijerph-1853482-supplementary.pdf]

Evaluation and Projection of Surface $PM_{2.5}$ and Its Exposure on Population in Asia Based on the CMIP6 GCMs

Ying Xu[1, 2, *], Jie Wu[3], Zhen-Yu Han[1, 2]

[1] National Climate Center, China Meteorological Administration, Beijing 100081, China

[2] Laboratory for Climate Studies, China Meteorological Administration, Beijing 100081, China

[3] School of Geography and Environmental Engineering, Gannan Normal University, Ganzhou 341000, China

14 Supplementary figures

Submitted to *International Journal of Environmental Research and Public Health*

July 22, 2022

Corresponding author:

Dr. Ying Xu, Email: xuying@cma.gov.cn

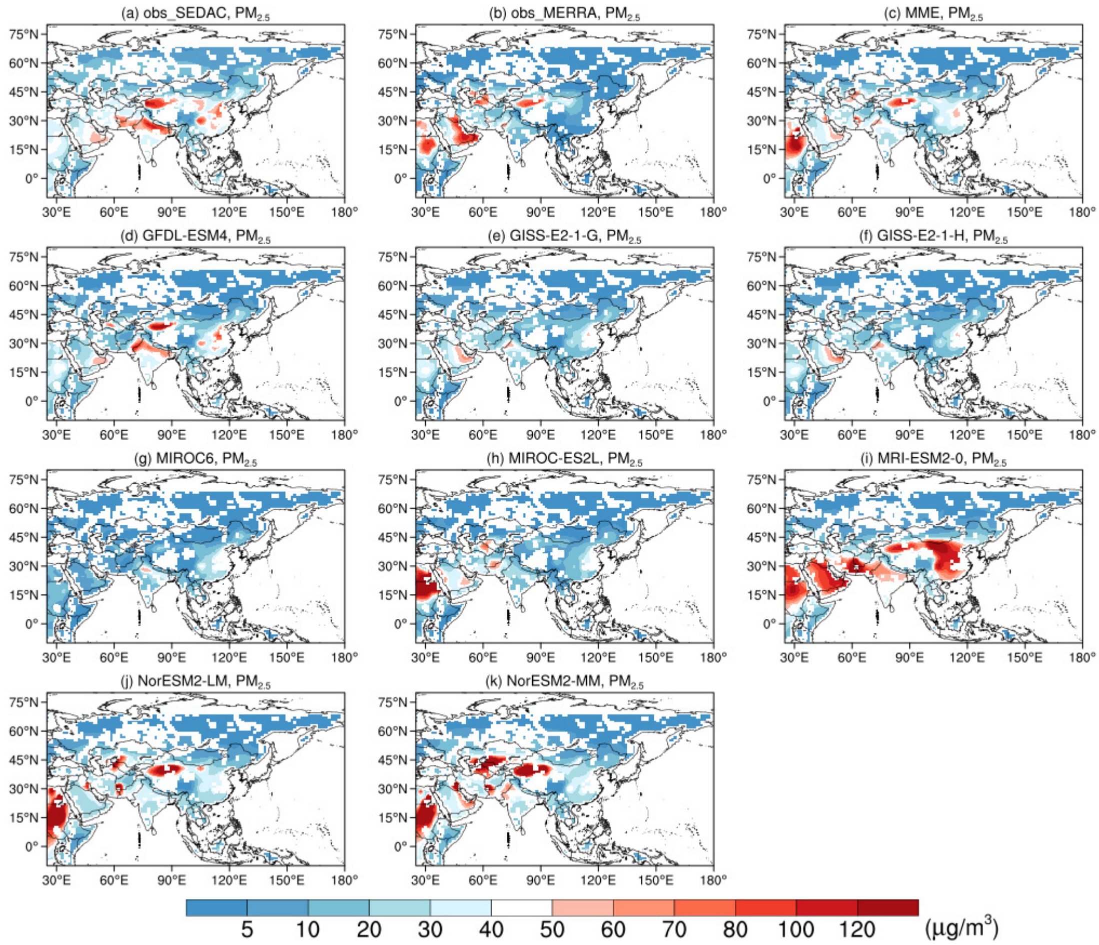

Figure S1. Spatial distribution of annual mean surface $PM_{2.5}$ concentrations (units: $\mu g/m^3$) during 1998-2014 from the observations, the original outputs of CMIP6 models and their bias.

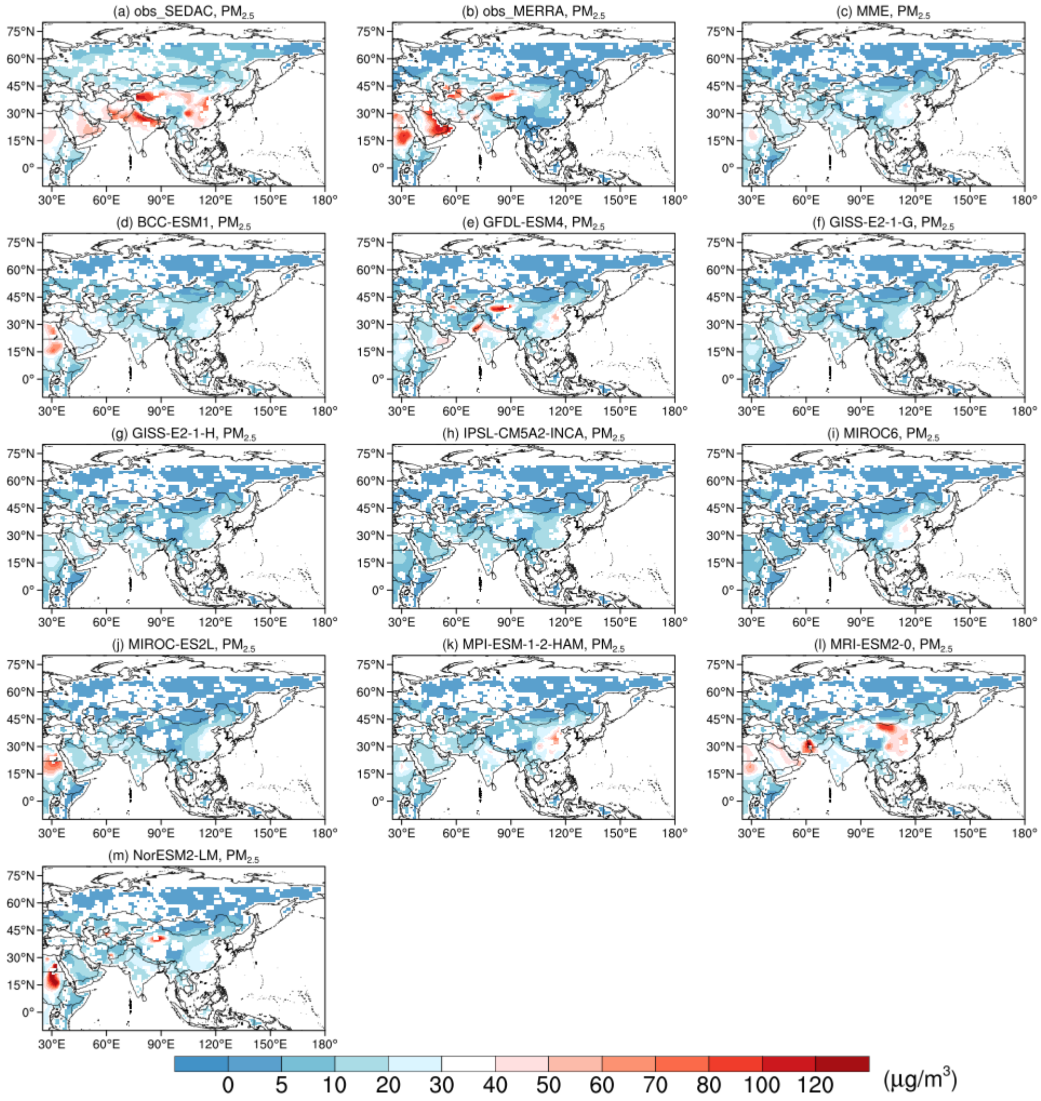

Figure S2. Same as Figure S1, but for the estimated.

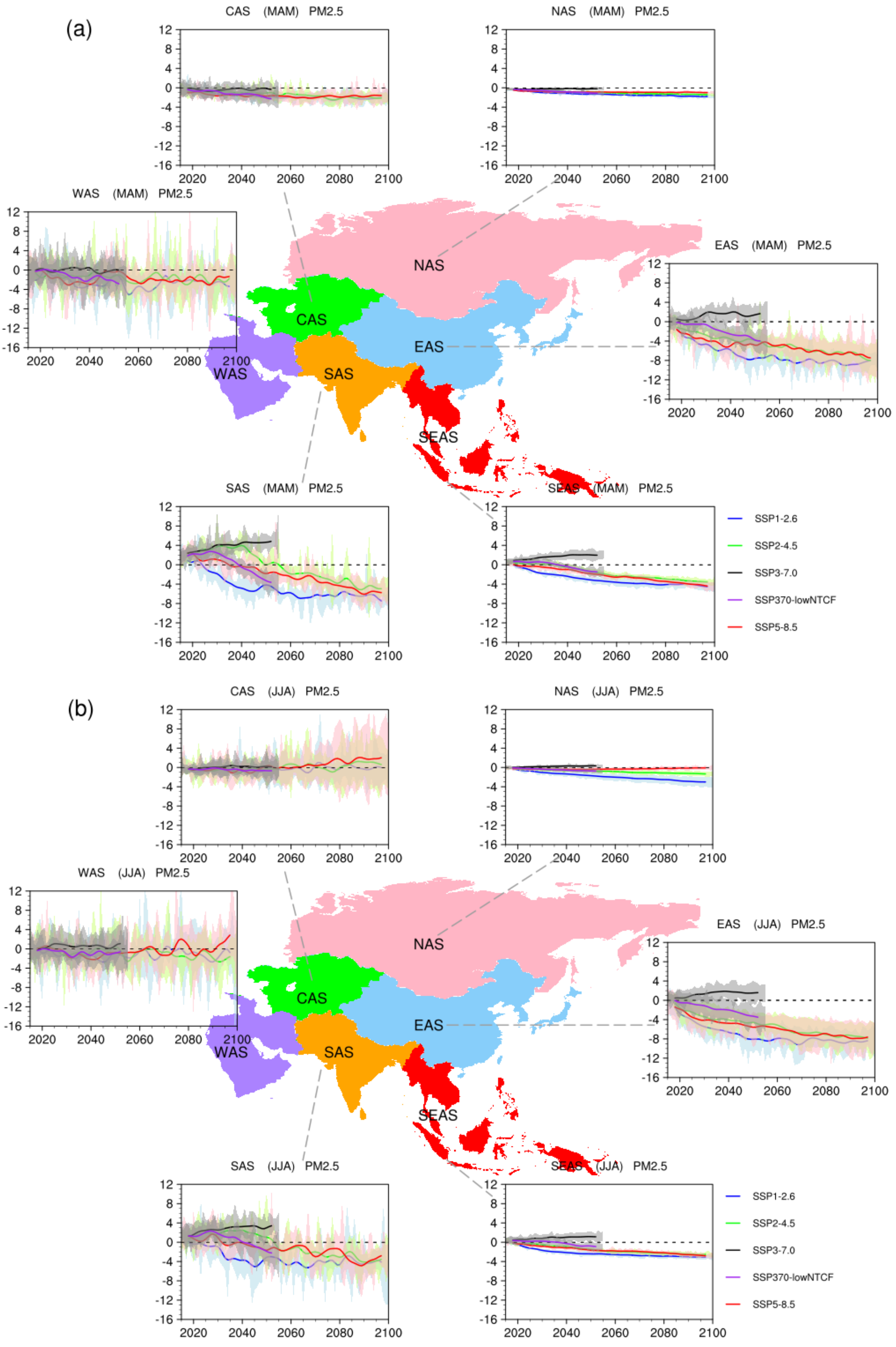
(a)
CAS (MAM) PM2.5
NAS (MAM) PM2.5
WAS (MAM) PM2.5
EAS (MAM) PM2.5
SAS (MAM) PM2.5
SEAS (MAM) PM2.5
NAS
CAS
EAS
WAS
SAS
SEAS
12
8
4
0
-4
-8
-12
-16
2020
2040
2060
2080
2100
SSP1-2.6
SSP2-4.5
SSP3-7.0
SSP370-lowNTCF
SSP5-8.5
(b)
CAS (JJA) PM2.5
NAS (JJA) PM2.5
WAS (JJA) PM2.5
EAS (JJA) PM2.5
SAS (JJA) PM2.5
SEAS (JJA) PM2.5

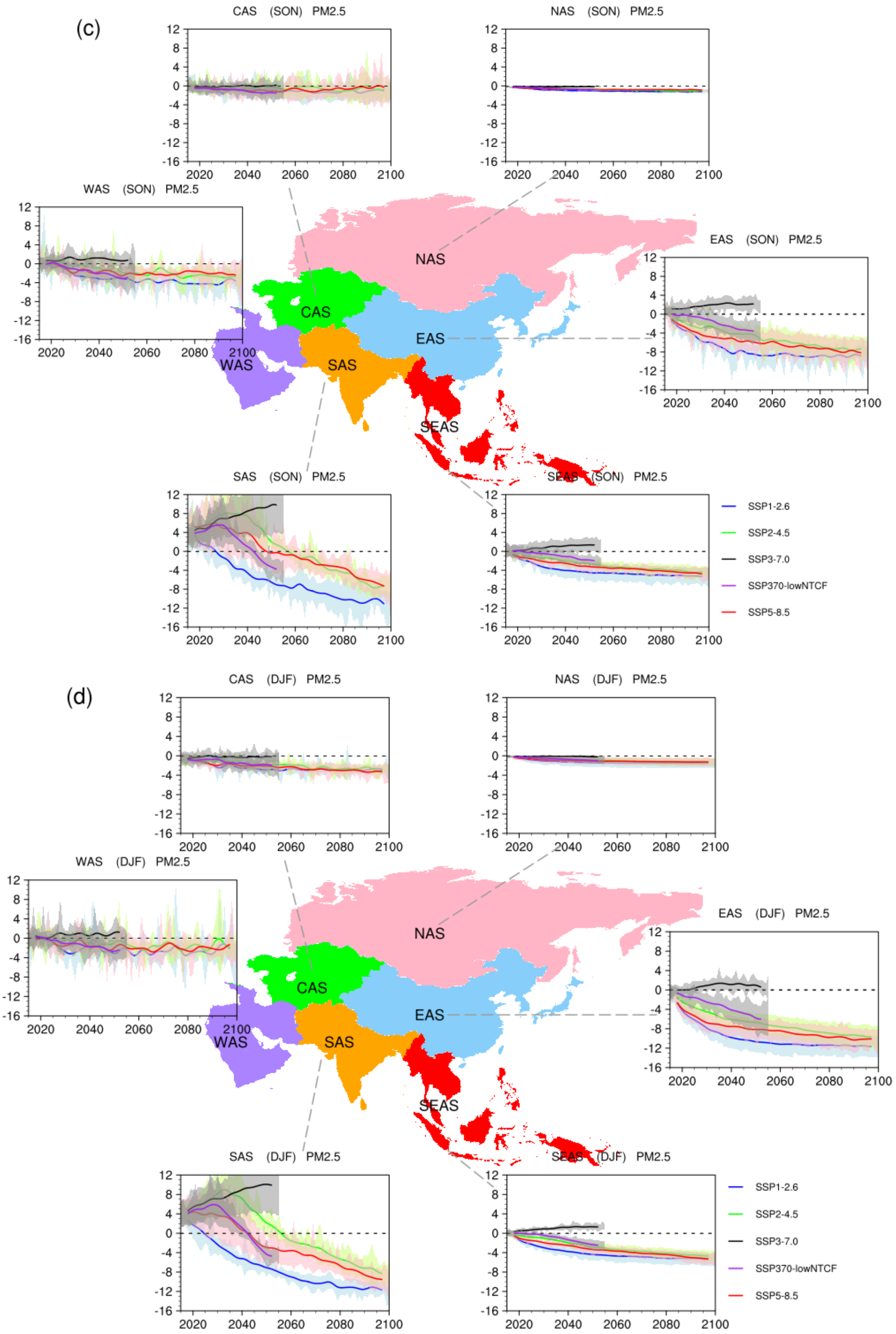

Figure S3 Time evolutions of surface $PM_{2.5}$ concentrations (units: $\mu g/m^3$) in spring(a), summer (b), autumn (c) and winter (d) over Asia under SSP1-2.6 (red curves), SSP2-4.5 (green curves), SSP3-7.0 (black curves), SSP370-lowNTCF (purple curves) and SSP5-8.5 (red curves), relative to 1995-2014, where shadings indicate model uncertainty range (1 standard deviation of the simulations).

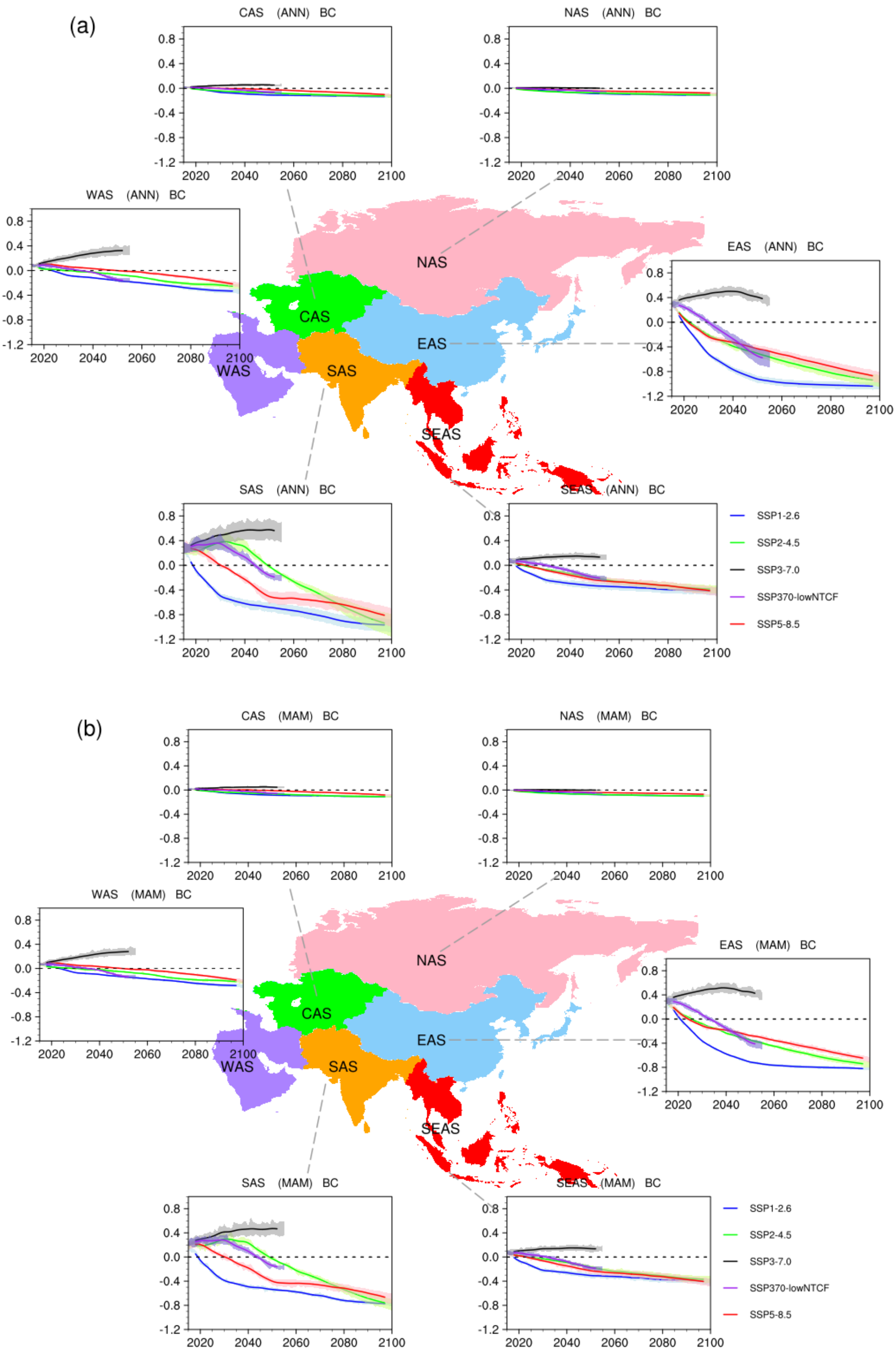
(a)
CAS (ANN) BC
NAS (ANN) BC
WAS (ANN) BC
EAS (ANN) BC
SAS (ANN) BC
SEAS (ANN) BC
NAS
CAS
EAS
WAS
SAS
SEAS
0.8
0.4
0.0
-0.4
-0.8
-1.2
2020
2040
2060
2080
2100
SSP1-2.6
SSP2-4.5
SSP3-7.0
SSP370-lowNTCF
SSP5-8.5
(b)
CAS (MAM) BC
NAS (MAM) BC
WAS (MAM) BC
EAS (MAM) BC
SAS (MAM) BC
SEAS (MAM) BC

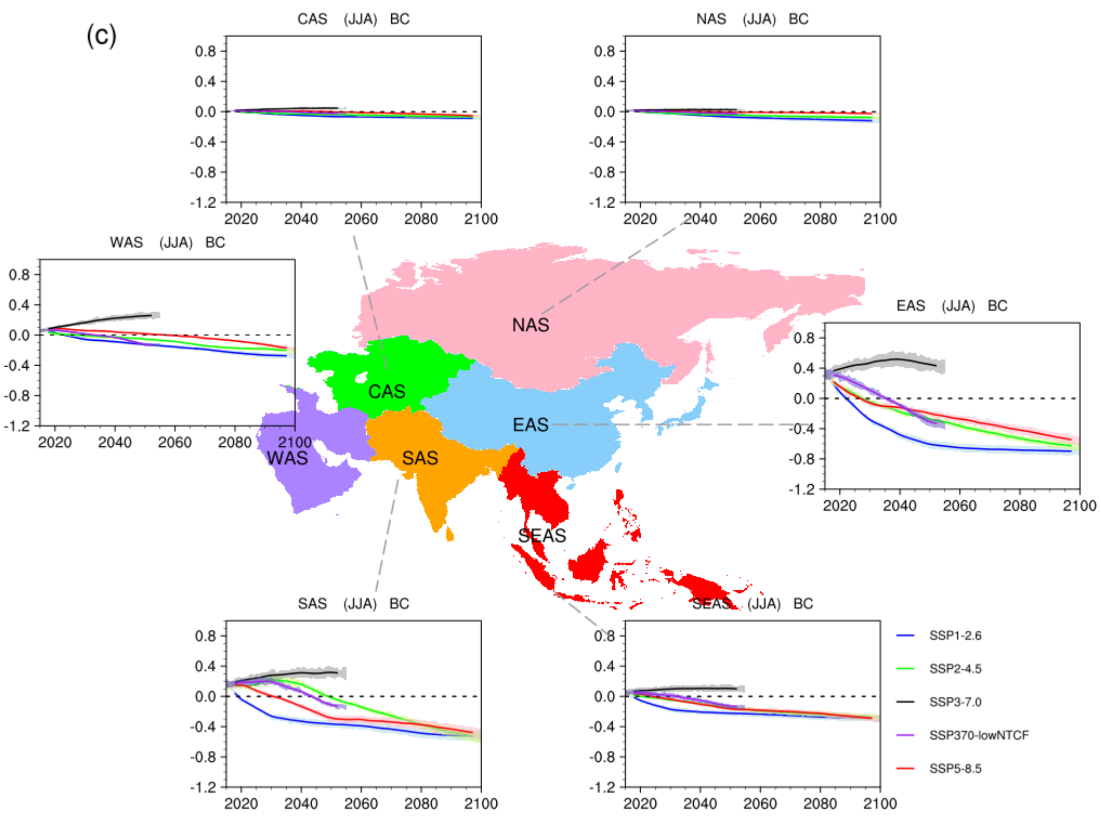
(c)
CAS (JJA) BC
NAS (JJA) BC
WAS (JJA) BC
EAS (JJA) BC
SAS (JJA) BC
SEAS (JJA) BC
NAS
CAS
EAS
WAS
SAS
SEAS
SSP1-2.6
SSP2-4.5
SSP3-7.0
SSP370-lowNTCF
SSP5-8.5

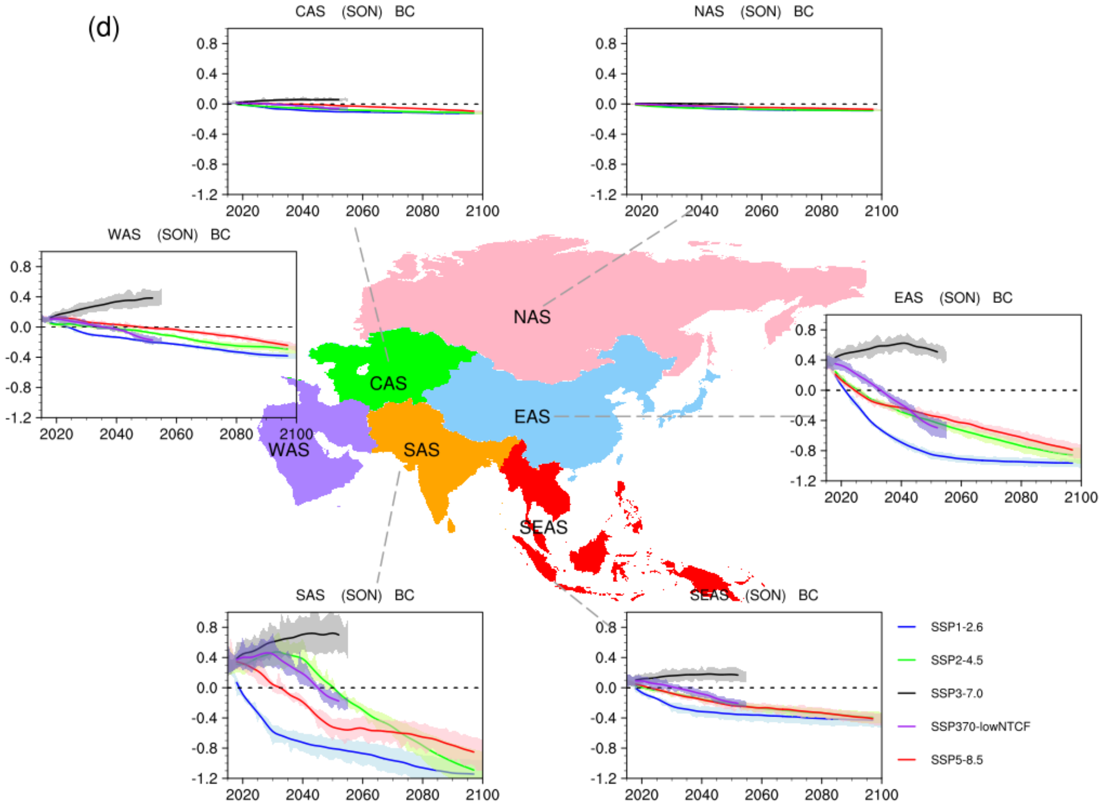
(d)
CAS (SON) BC
NAS (SON) BC
WAS (SON) BC
EAS (SON) BC
SAS (SON) BC
SEAS (SON) BC
NAS
CAS
EAS
WAS
SAS
SEAS
SSP1-2.6
SSP2-4.5
SSP3-7.0
SSP370-lowNTCF
SSP5-8.5

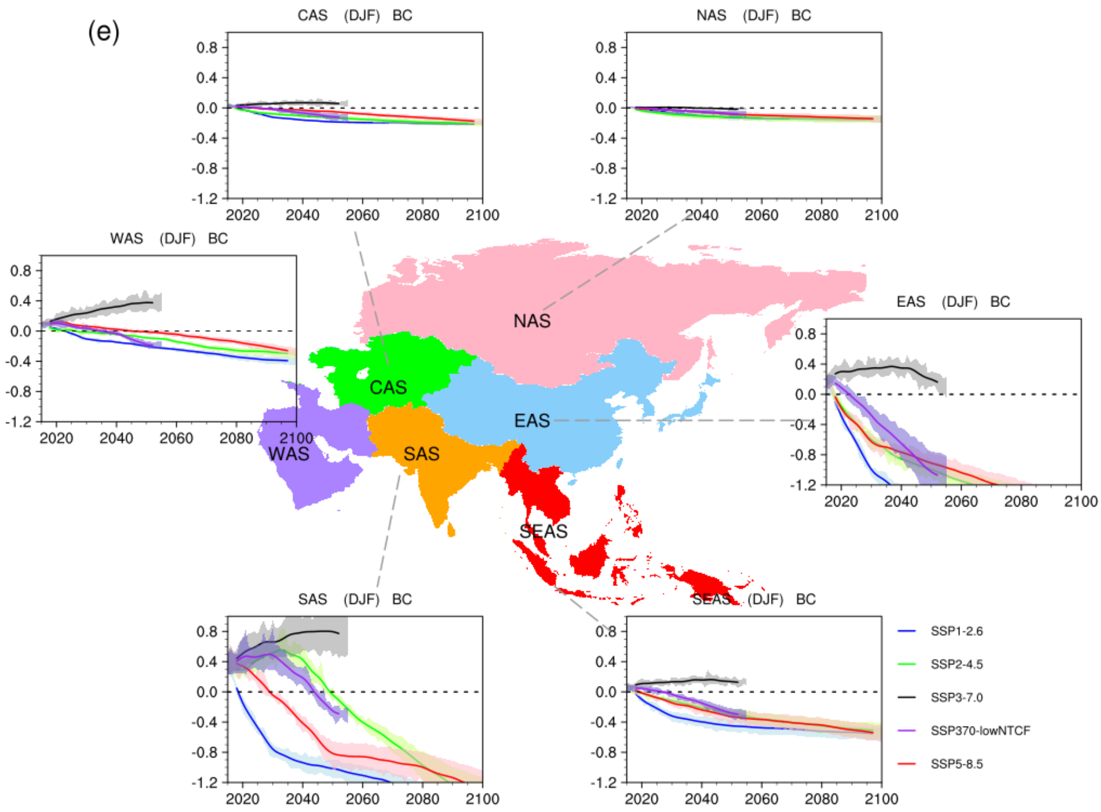

Figure S4 Time evolutions of surface black carbon (BC) concentrations (units: $\mu g/m^3$) in annual (a), spring (b), summer (c), autumn (d) and winter (e) over Asia under SSP1-2.6 (red curves), SSP2-4.5 (green curves), SSP3-7.0 (black curves), SSP370-lowNTCF (purple curves) and SSP5-8.5 (red curves), relative to 1995-2014, where shadings indicate model uncertainty range (1 standard deviation of the simulations).

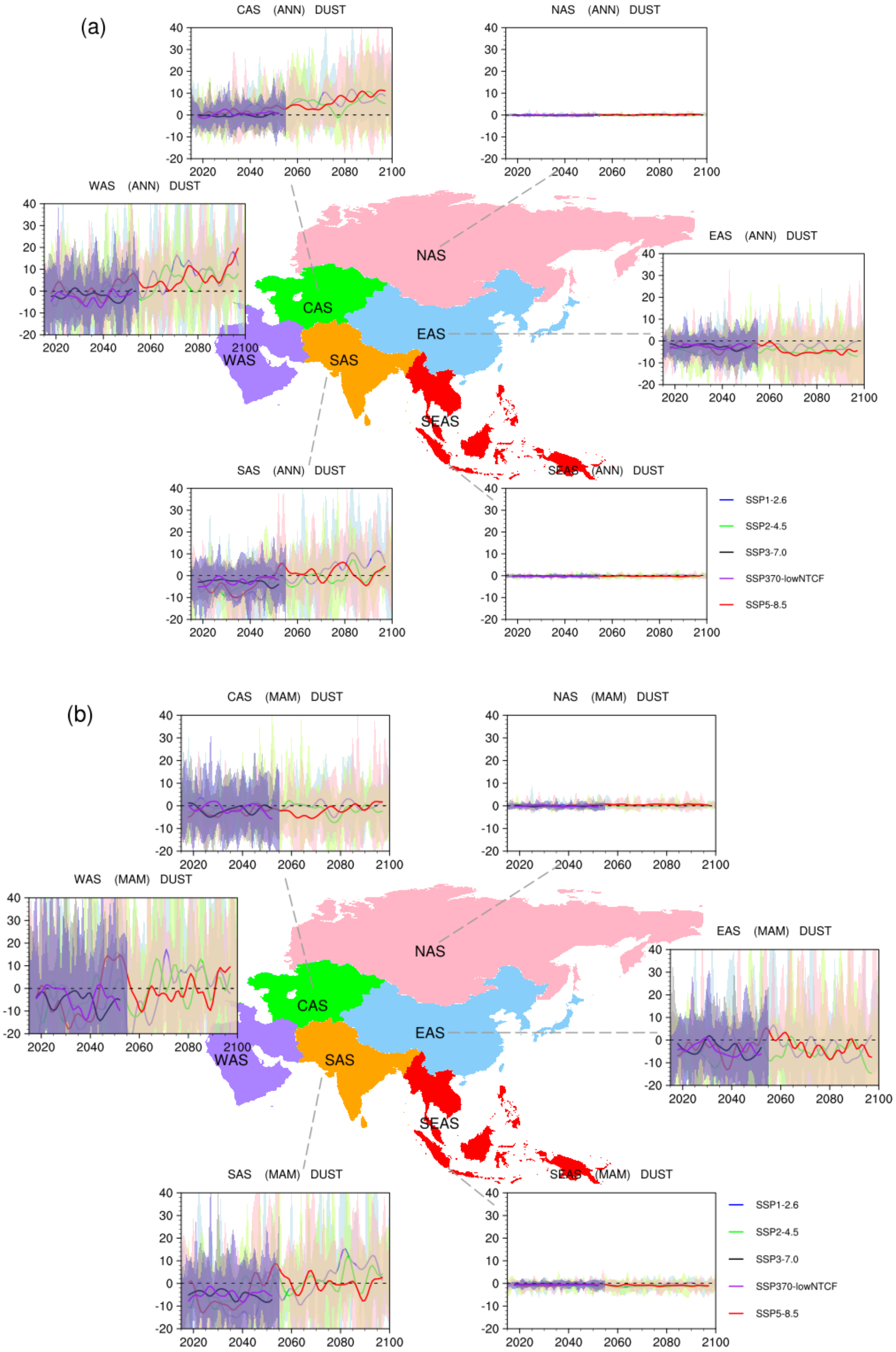
(a)
CAS (ANN) DUST
NAS (ANN) DUST
WAS (ANN) DUST
EAS (ANN) DUST
SAS (ANN) DUST
SEAS (ANN) DUST
NAS
CAS
EAS
WAS
SAS
SEAS
SSP1-2.6
SSP2-4.5
SSP3-7.0
SSP370-lowNTCF
SSP5-8.5
(b)
CAS (MAM) DUST
NAS (MAM) DUST
WAS (MAM) DUST
EAS (MAM) DUST
SAS (MAM) DUST
SEAS (MAM) DUST
NAS
CAS
EAS
WAS
SAS
SEAS
SSP1-2.6
SSP2-4.5
SSP3-7.0
SSP370-lowNTCF
SSP5-8.5

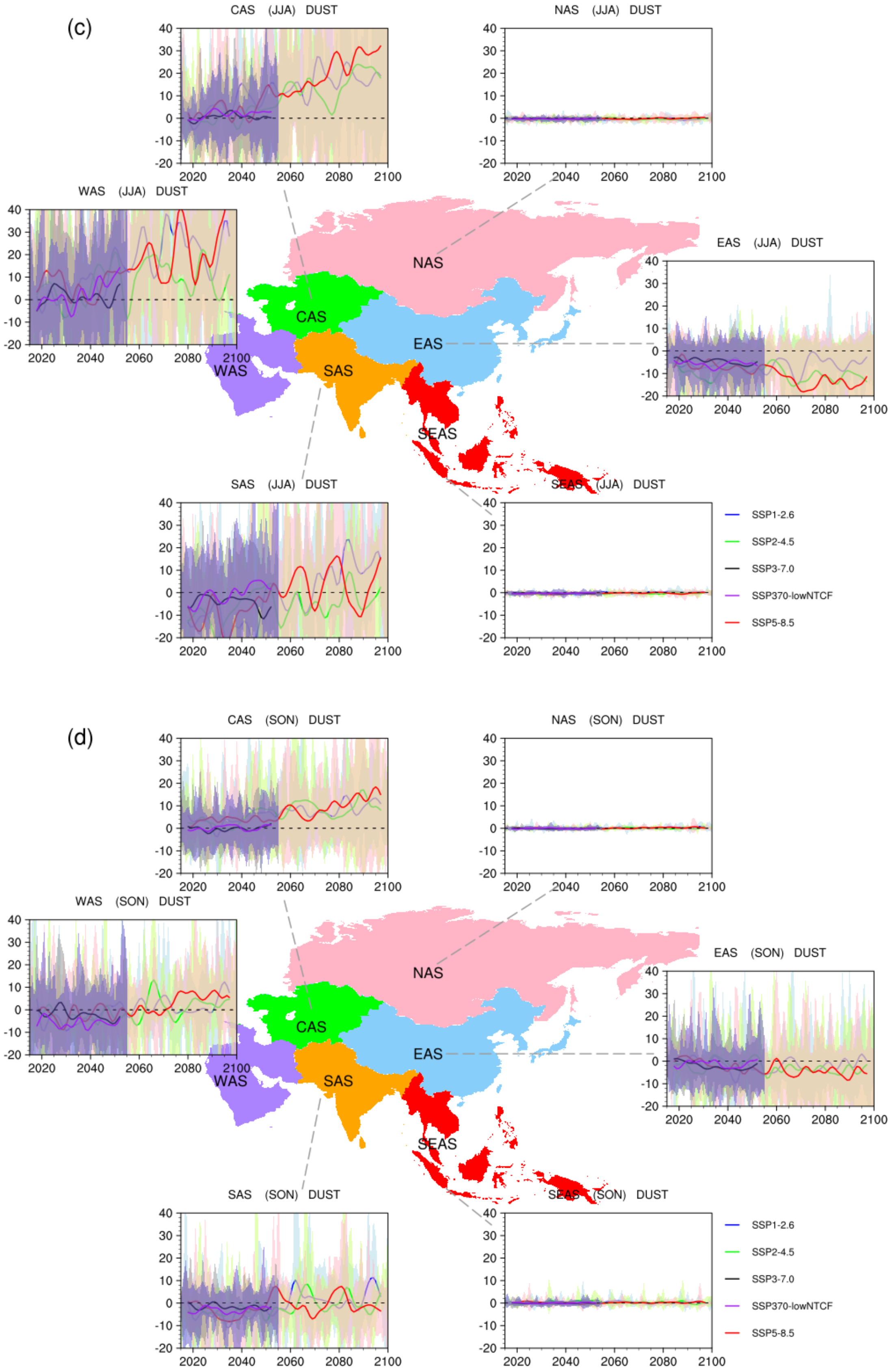

(c)
CAS (JJA) DUST
NAS (JJA) DUST
WAS (JJA) DUST
EAS (JJA) DUST
SAS (JJA) DUST
SEAS (JJA) DUST
NAS
CAS
EAS
WAS
SAS
SEAS
SSP1-2.6
SSP2-4.5
SSP3-7.0
SSP370-lowNTCF
SSP5-8.5
(d)
CAS (SON) DUST
NAS (SON) DUST
WAS (SON) DUST
EAS (SON) DUST
SAS (SON) DUST
SEAS (SON) DUST
NAS
CAS
EAS
WAS
SAS
SEAS
SSP1-2.6
SSP2-4.5
SSP3-7.0
SSP370-lowNTCF
SSP5-8.5

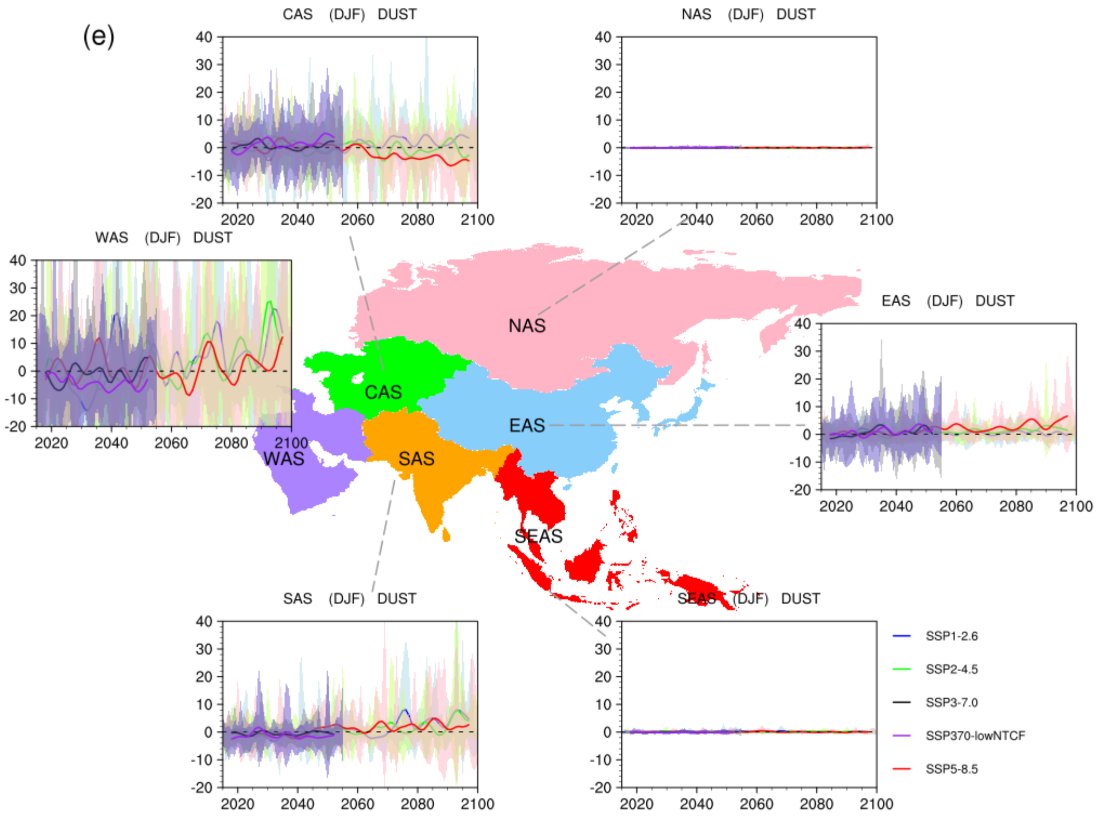

Figure S5 Time evolutions of surface dust concentrations (units: $\mu g/m^3$) in annual (a), spring (b), summer(c), autumn (d) and winter (e) over Asia under SSP1-2.6 (red curves), SSP2-4.5 (green curves), SSP3-7.0 (black curves), SSP370-lowNTCF (purple curves) and SSP5-8.5 (red curves), relative to 1995-2014, where shadings indicate model uncertainty range (1 standard deviation of the simulations).

.

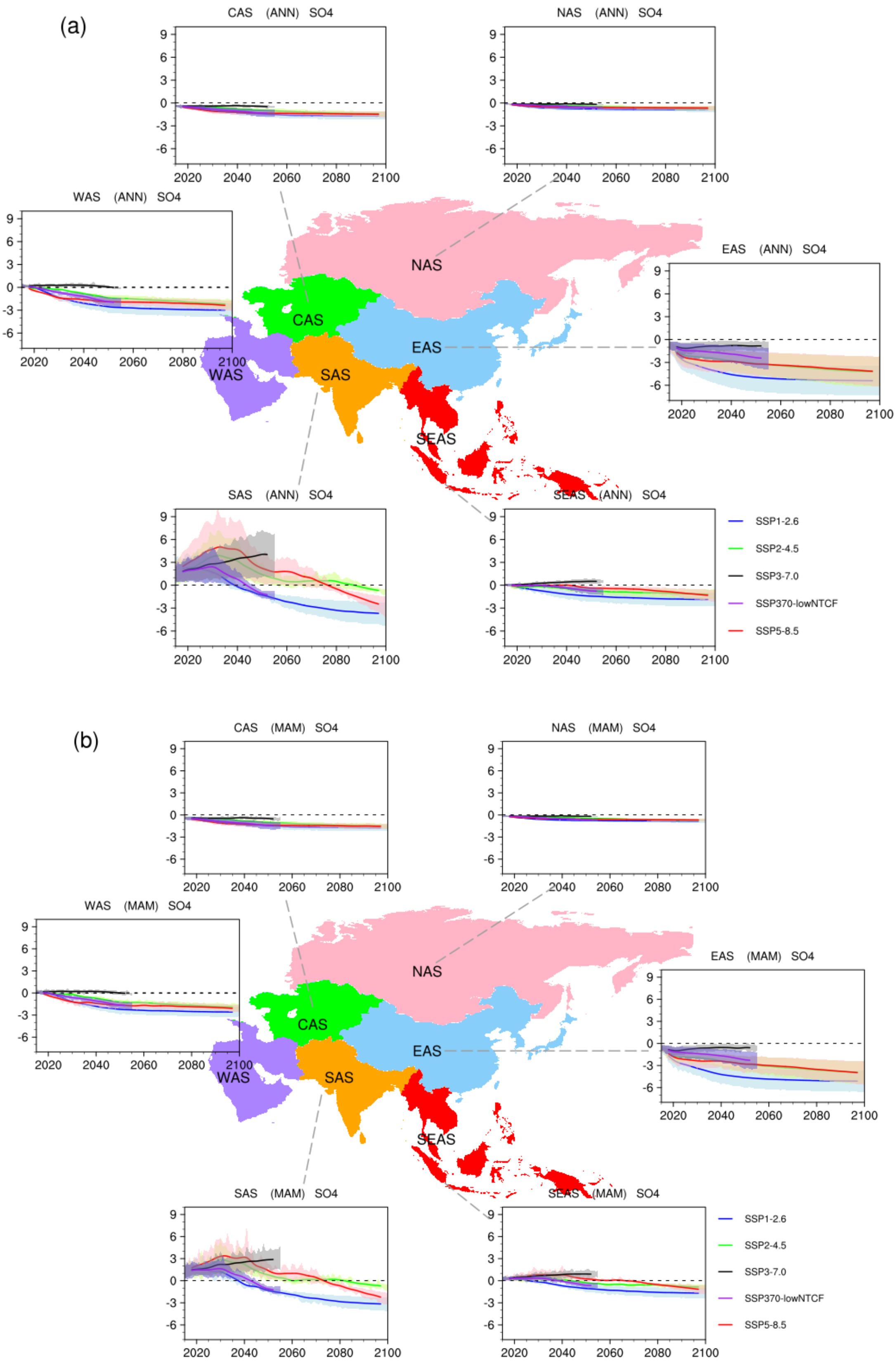
(a)
CAS (ANN) SO4
NAS (ANN) SO4
WAS (ANN) SO4
EAS (ANN) SO4
SAS (ANN) SO4
SEAS (ANN) SO4
NAS
CAS
EAS
WAS
SAS
SEAS
SSP1-2.6
SSP2-4.5
SSP3-7.0
SSP370-lowNTCF
SSP5-8.5
(b)
CAS (MAM) SO4
NAS (MAM) SO4
WAS (MAM) SO4
EAS (MAM) SO4
SAS (MAM) SO4
SEAS (MAM) SO4
NAS
CAS
EAS
WAS
SAS
SEAS
SSP1-2.6
SSP2-4.5
SSP3-7.0
SSP370-lowNTCF
SSP5-8.5

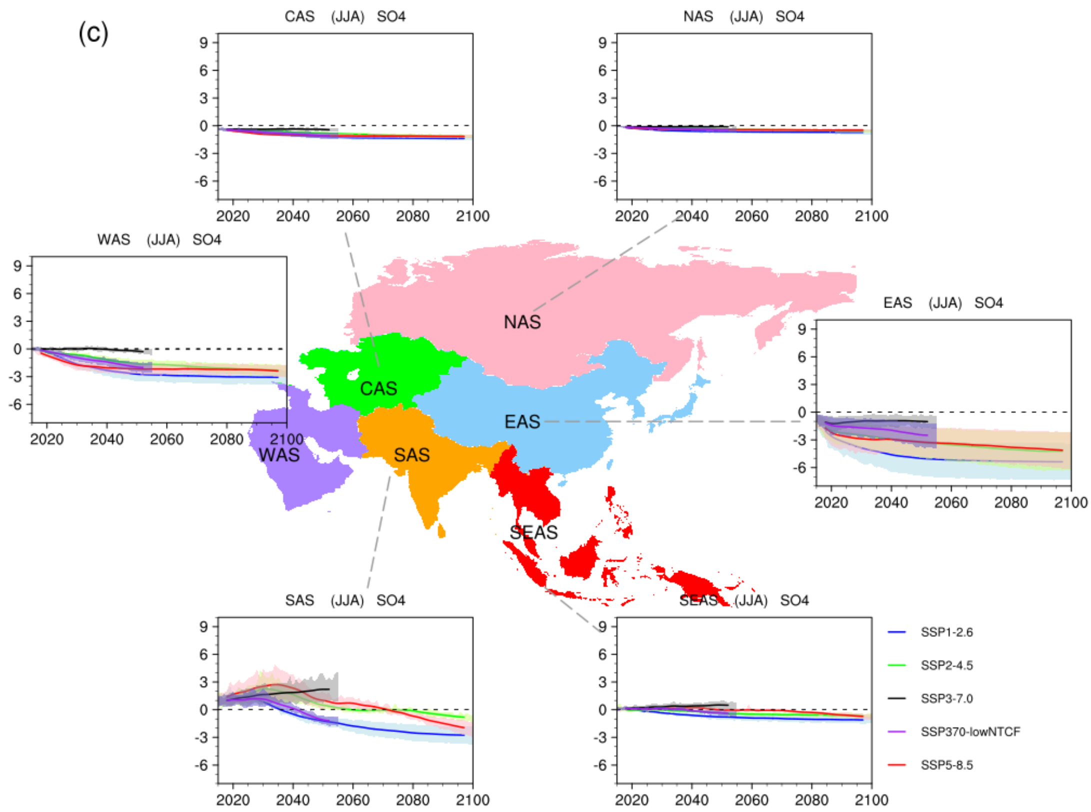
(c)
CAS (JJA) SO4
NAS (JJA) SO4
WAS (JJA) SO4
EAS (JJA) SO4
SAS (JJA) SO4
SEAS (JJA) SO4
NAS
CAS
EAS
WAS
SAS
SEAS
SSP1-2.6
SSP2-4.5
SSP3-7.0
SSP370-lowNTCF
SSP5-8.5

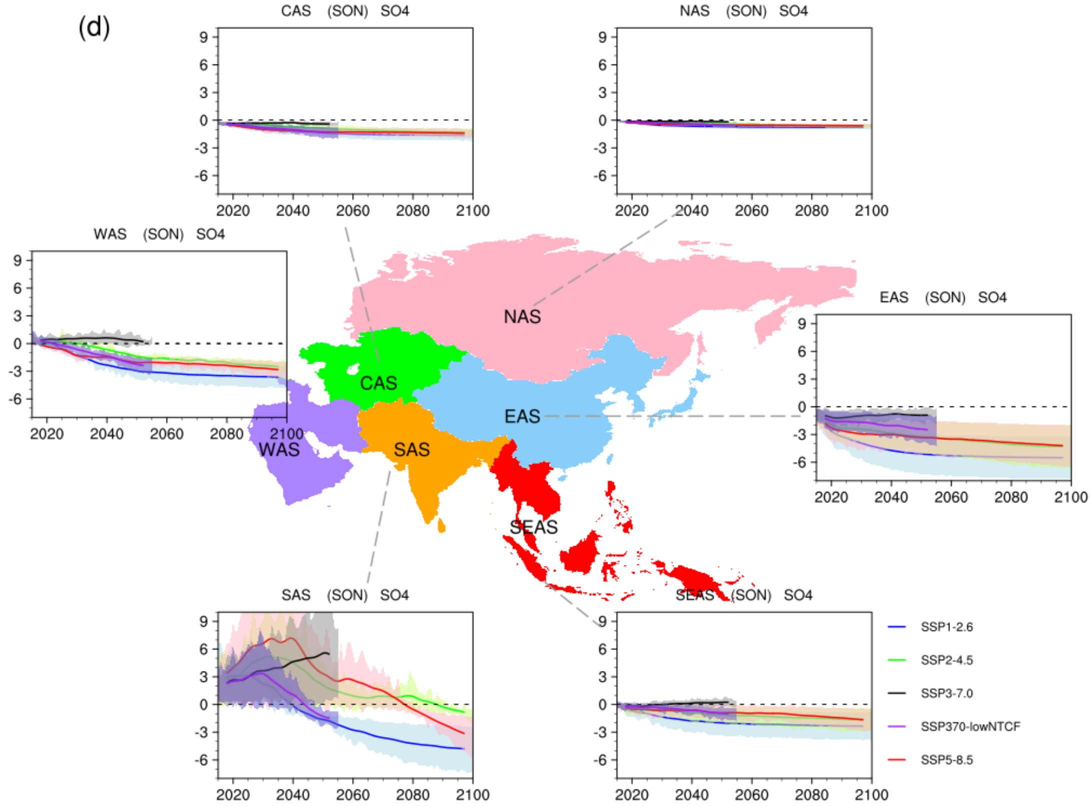
(d)
CAS (SON) SO4
NAS (SON) SO4
WAS (SON) SO4
EAS (SON) SO4
SAS (SON) SO4
SEAS (SON) SO4
NAS
CAS
EAS
WAS
SAS
SEAS
SSP1-2.6
SSP2-4.5
SSP3-7.0
SSP370-lowNTCF
SSP5-8.5

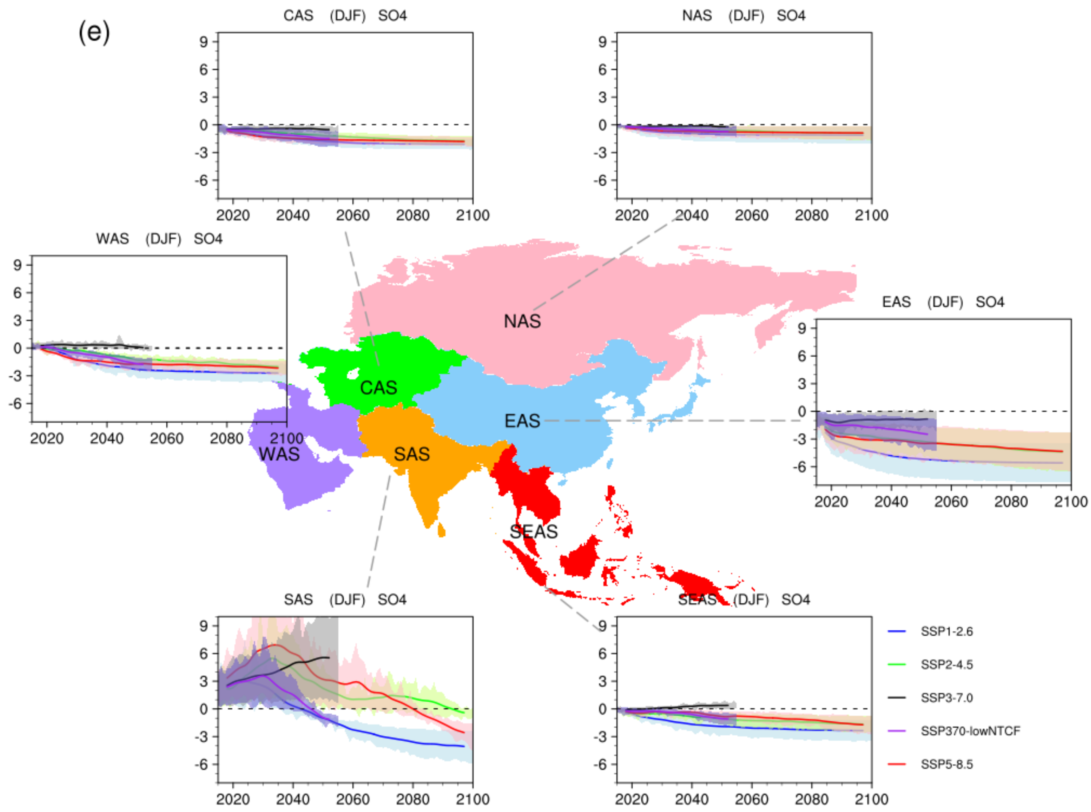

Figure S6 Time evolutions of surface SO_4 concentrations (units: $\mu g/m^3$) in annual (a), spring (b), summer (c), autumn (d) and winter (e) over Asia under SSP1-2.6 (red curves), SSP2-4.5 (green curves), SSP3-7.0 (black curves), SSP370-lowNTCF (purple curves) and SSP5-8.5 (red curves), relative to 1995-2014, where shadings indicate model uncertainty range (1 standard deviation of the simulations).

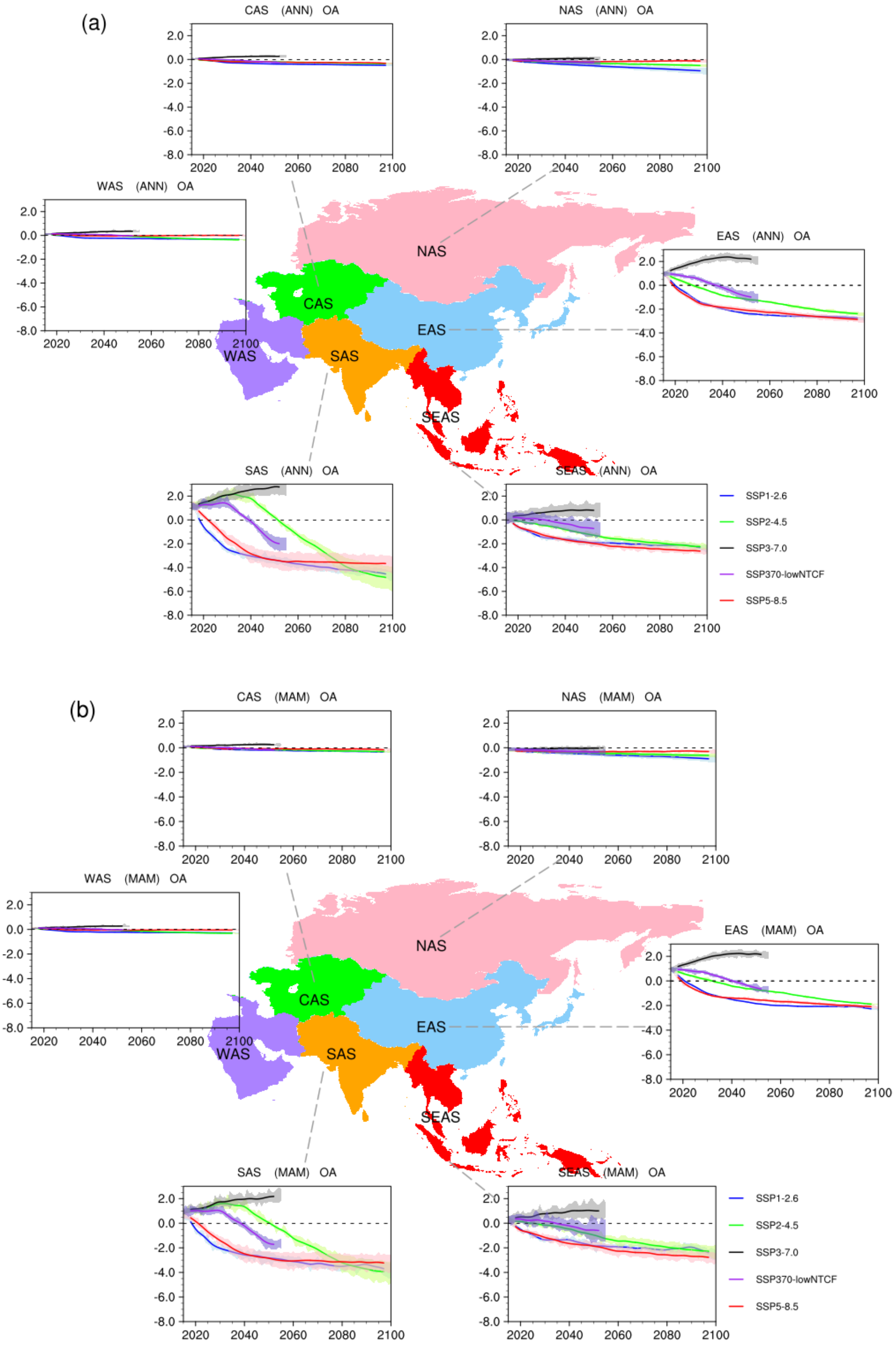
(a)
CAS (ANN) OA
NAS (ANN) OA
WAS (ANN) OA
EAS (ANN) OA
SAS (ANN) OA
SEAS (ANN) OA
2.0
0.0
-2.0
-4.0
-6.0
-8.0
2020
2040
2060
2080
2100
NAS
CAS
EAS
WAS
SAS
SEAS
SSP1-2.6
SSP2-4.5
SSP3-7.0
SSP370-lowNTCF
SSP5-8.5
(b)
CAS (MAM) OA
NAS (MAM) OA
WAS (MAM) OA
EAS (MAM) OA
SAS (MAM) OA
SEAS (MAM) OA

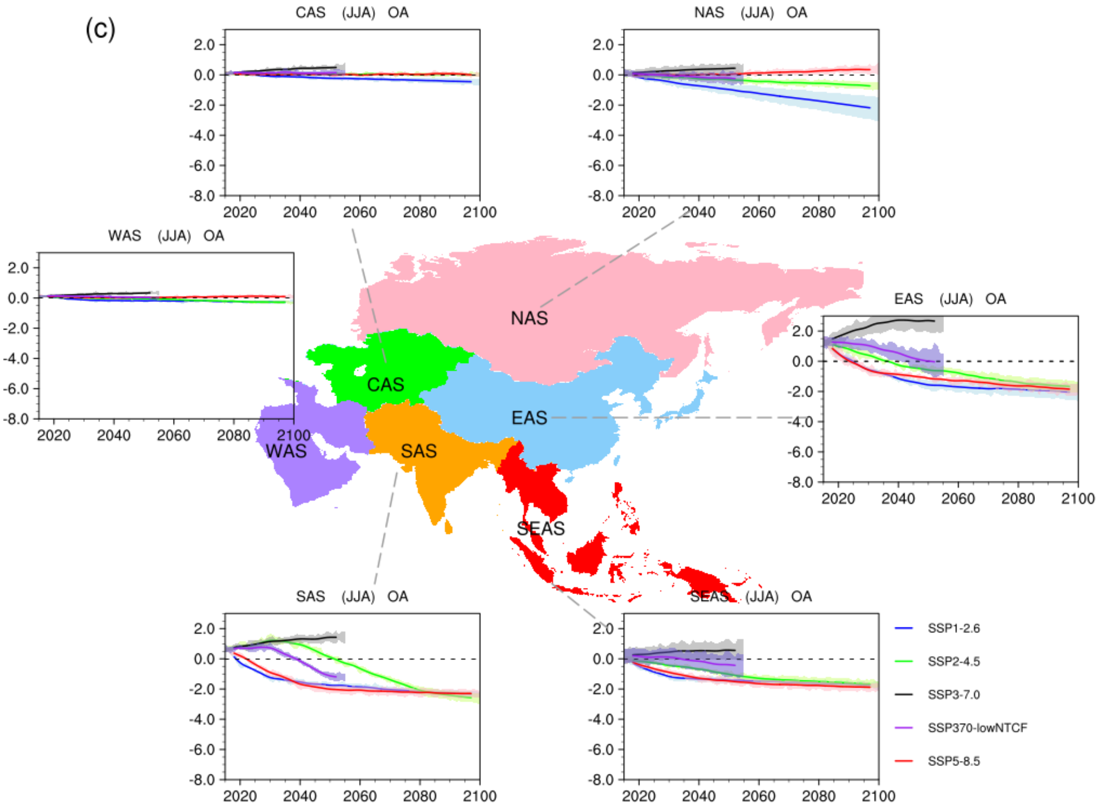

(c)
CAS (JJA) OA
NAS (JJA) OA
WAS (JJA) OA
EAS (JJA) OA
SAS (JJA) OA
SEAS (JJA) OA
NAS
CAS
EAS
WAS
SAS
SEAS
SSP1-2.6
SSP2-4.5
SSP3-7.0
SSP370-lowNTCF
SSP5-8.5

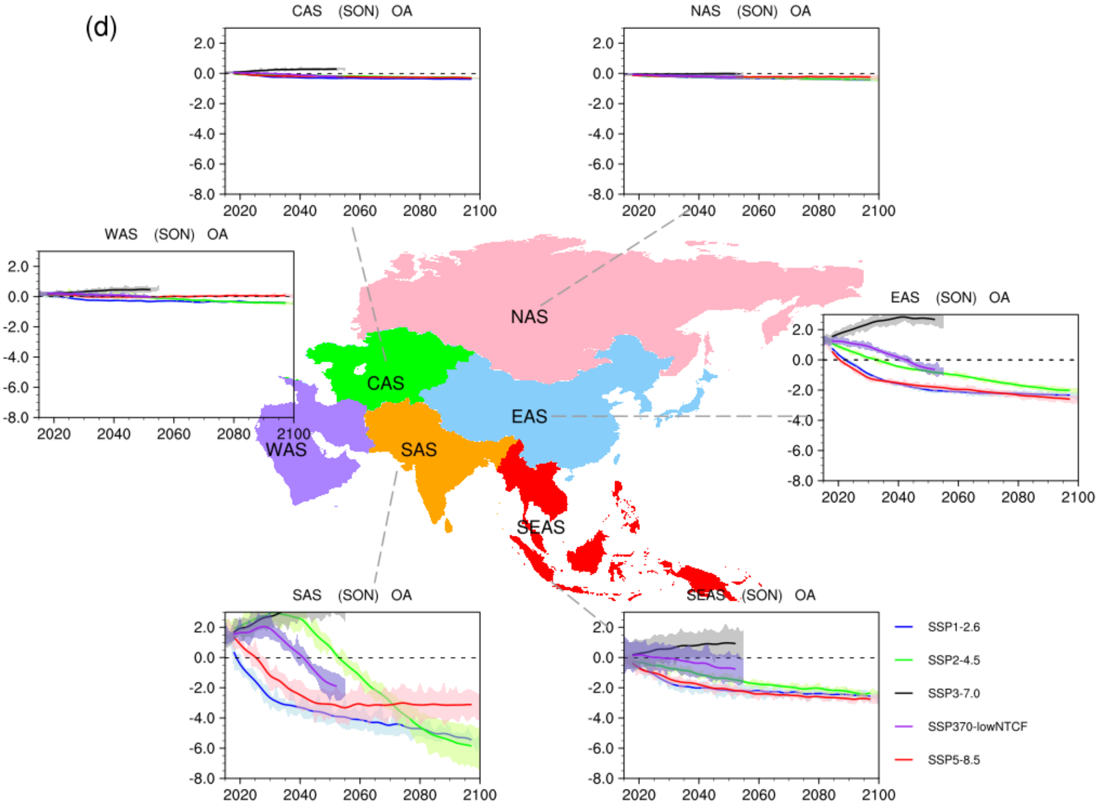

(d)
CAS (SON) OA
NAS (SON) OA
WAS (SON) OA
EAS (SON) OA
SAS (SON) OA
SEAS (SON) OA
NAS
CAS
EAS
WAS
SAS
SEAS
SSP1-2.6
SSP2-4.5
SSP3-7.0
SSP370-lowNTCF
SSP5-8.5

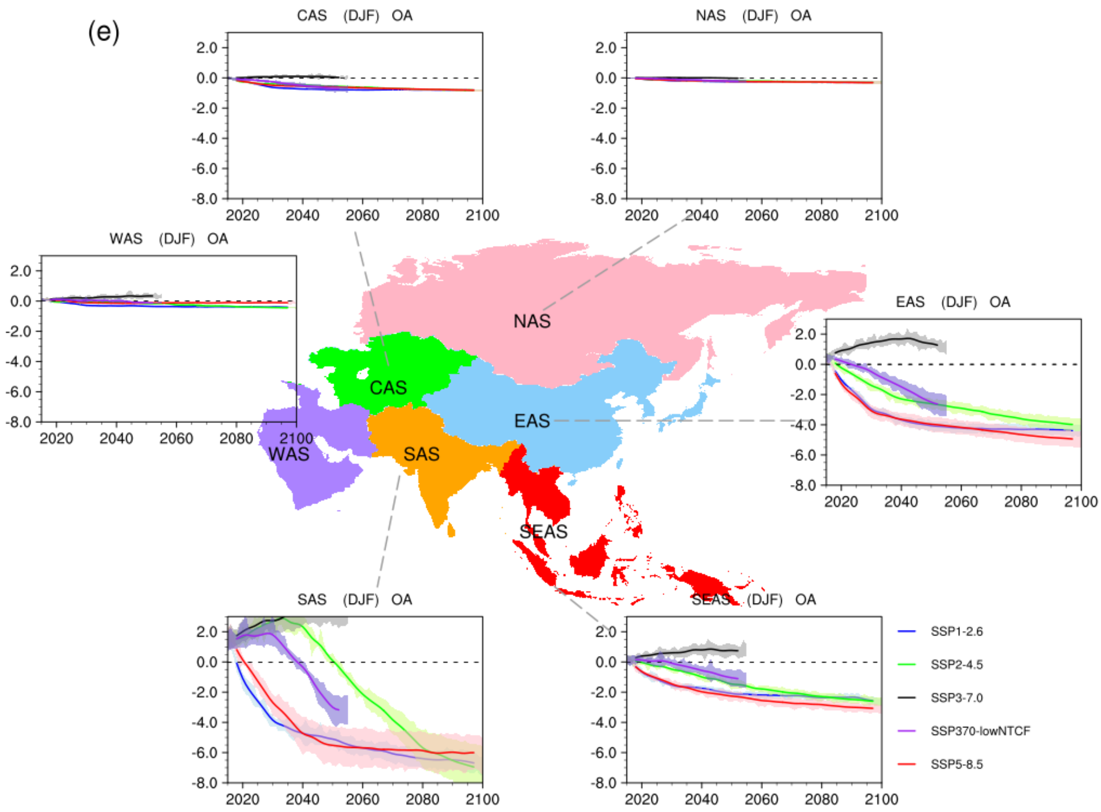

Figure S7 Time evolutions of surface organic aerosol (OA) concentrations (units: $\mu g/m^3$) in annual (a), spring (b), summer(c), autumn (d) and winter (e) over Asia under SSP1-2.6 (red curves), SSP2-4.5 (green curves), SSP3-7.0 (black curves), SSP370-lowNTCF (purple curves) and SSP5-8.5 (red curves), relative to 1995-2014, where shadings indicate model uncertainty range (1 standard deviation of the simulations).

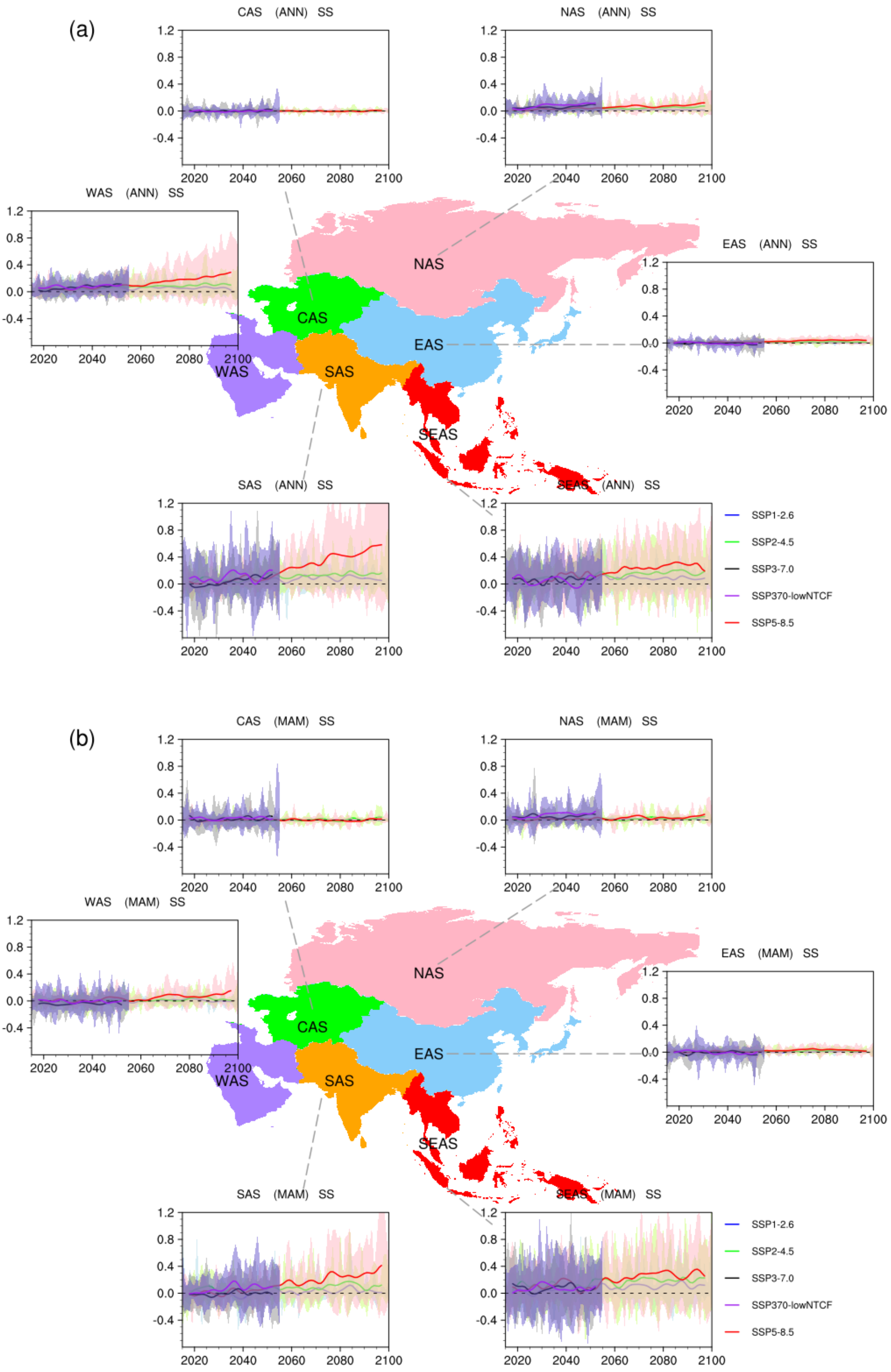
(a)
CAS (ANN) SS
NAS (ANN) SS
WAS (ANN) SS
EAS (ANN) SS
SAS (ANN) SS
SEAS (ANN) SS
NAS
CAS
EAS
WAS
SAS
SEAS
SSP1-2.6
SSP2-4.5
SSP3-7.0
SSP370-lowNTCF
SSP5-8.5
(b)
CAS (MAM) SS
NAS (MAM) SS
WAS (MAM) SS
EAS (MAM) SS
SAS (MAM) SS
SEAS (MAM) SS
NAS
CAS
EAS
WAS
SAS
SEAS
SSP1-2.6
SSP2-4.5
SSP3-7.0
SSP370-lowNTCF
SSP5-8.5

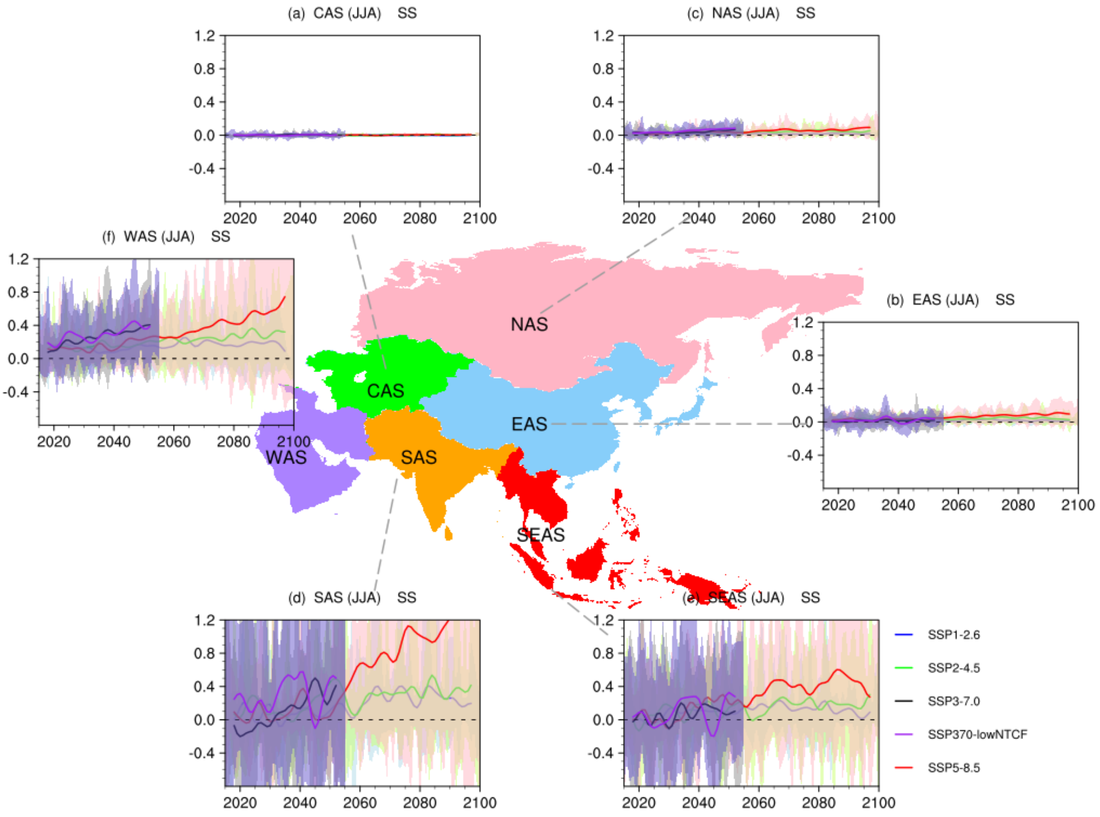
(a) CAS (JJA) SS
(c) NAS (JJA) SS
(f) WAS (JJA) SS
(b) EAS (JJA) SS
(d) SAS (JJA) SS
(e) SEAS (JJA) SS
NAS
CAS
EAS
WAS
SAS
SEAS
SSP1-2.6
SSP2-4.5
SSP3-7.0
SSP370-lowNTCF
SSP5-8.5

(d)

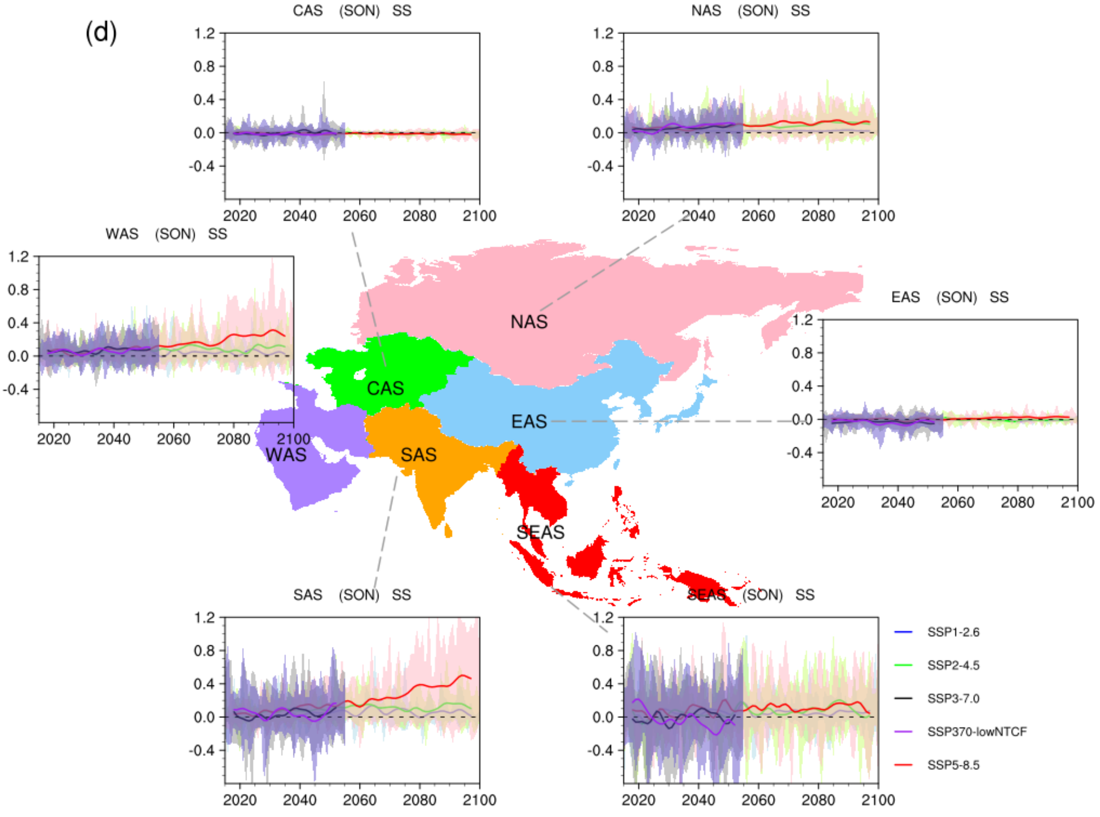
CAS (SON) SS
NAS (SON) SS
WAS (SON) SS
EAS (SON) SS
SAS (SON) SS
SEAS (SON) SS
NAS
CAS
EAS
WAS
SAS
SEAS
SSP1-2.6
SSP2-4.5
SSP3-7.0
SSP370-lowNTCF
SSP5-8.5

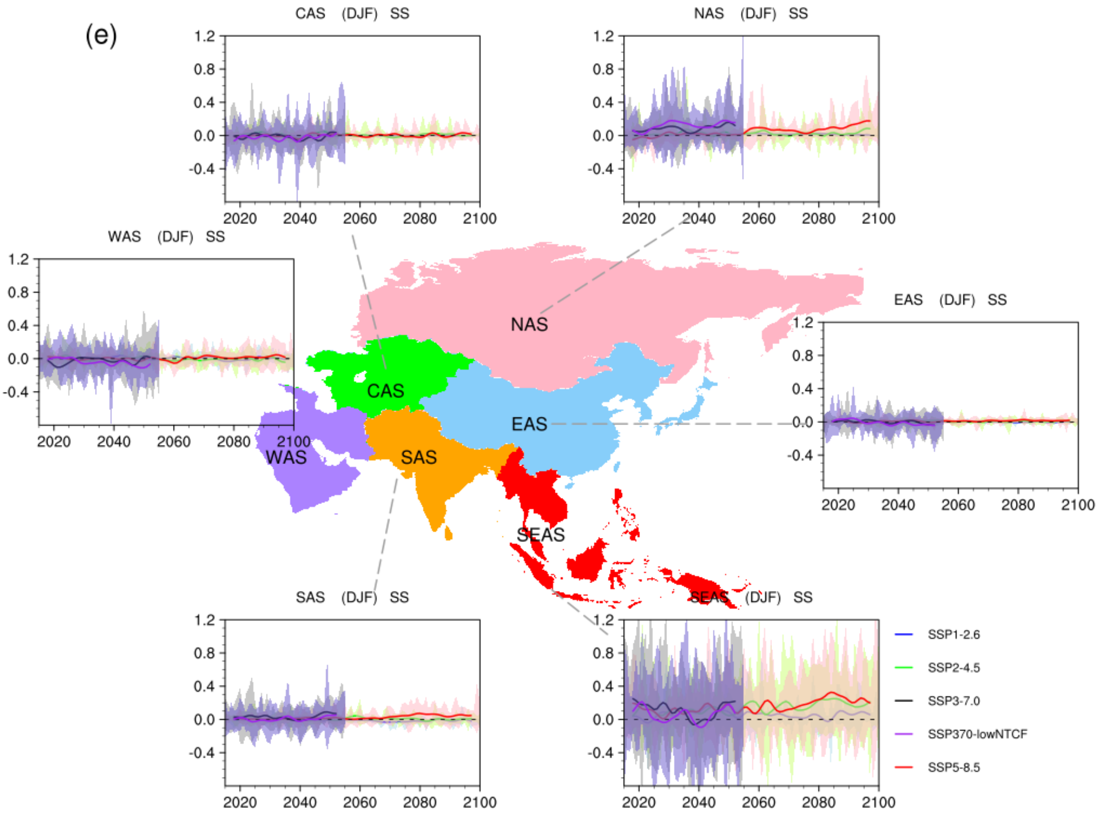

Figure S8 Time evolutions of surface sea salt (SS) concentrations (units: $\mu g/m^3$) in annual (a), spring (b), summer (c), autumn (d) and winter (e) over Asia under SSP1-2.6 (red curves), SSP2-4.5 (green curves), SSP3-7.0 (black curves), SSP370-lowNTCF (purple curves) and SSP5-8.5 (red curves), relative to 1995-2014, where shadings indicate model uncertainty range (1 standard deviation of the simulations).

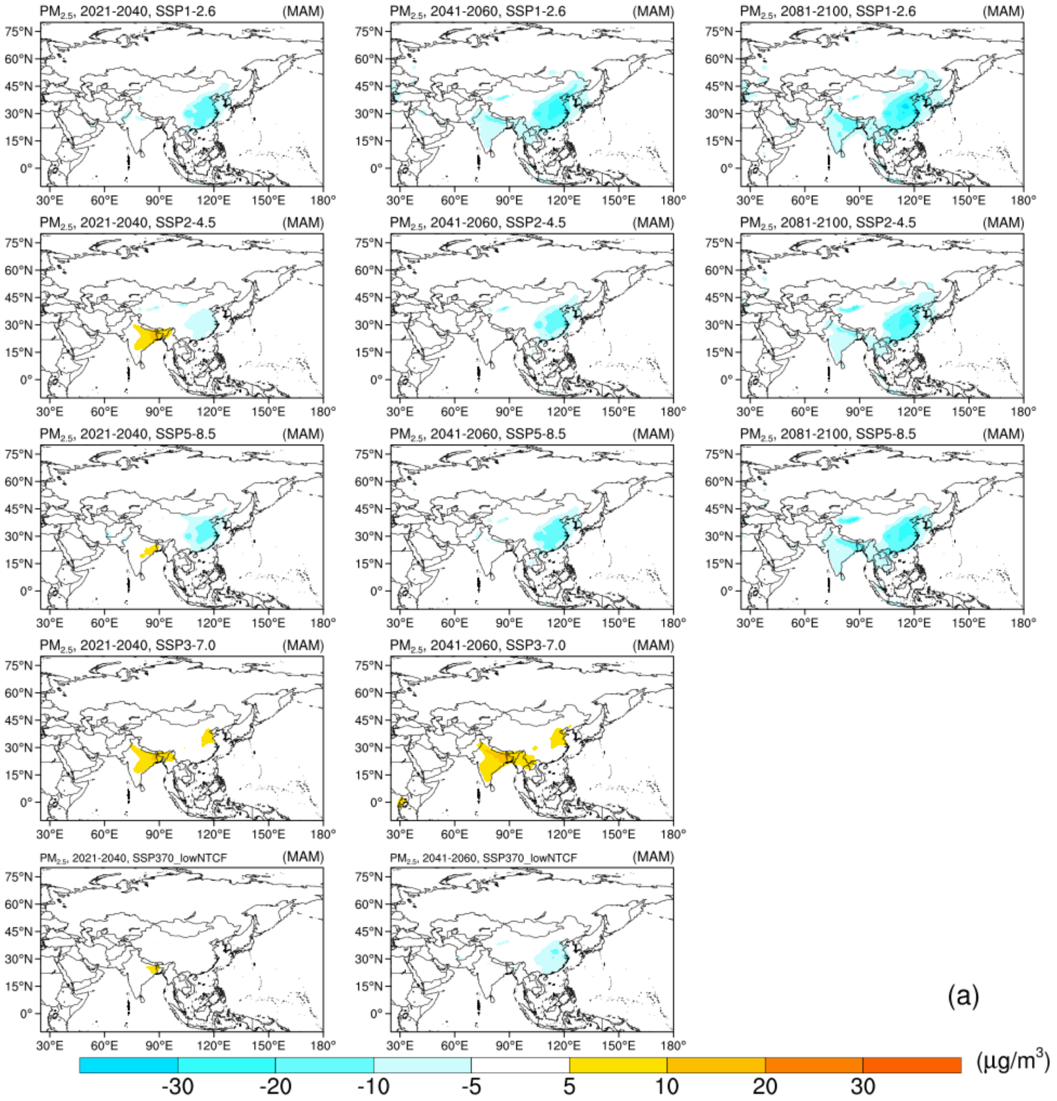

PM2.5, 2021-2040, SSP1-2.6 (MAM)
PM2.5, 2041-2060, SSP1-2.6 (MAM)
PM2.5, 2081-2100, SSP1-2.6 (MAM)
PM2.5, 2021-2040, SSP2-4.5 (MAM)
PM2.5, 2041-2060, SSP2-4.5 (MAM)
PM2.5, 2081-2100, SSP2-4.5 (MAM)
PM2.5, 2021-2040, SSP5-8.5 (MAM)
PM2.5, 2041-2060, SSP5-8.5 (MAM)
PM2.5, 2081-2100, SSP5-8.5 (MAM)
PM2.5, 2021-2040, SSP3-7.0 (MAM)
PM2.5, 2041-2060, SSP3-7.0 (MAM)
PM2.5, 2021-2040, SSP370_lowNTCF (MAM)
PM2.5, 2041-2060, SSP370_lowNTCF (MAM)
75°N 60°N 45°N 30°N 15°N 0°
30°E 60°E 90°E 120°E 150°E 180°
(a)
-30 -20 -10 -5 5 10 20 30
(μg/m3)

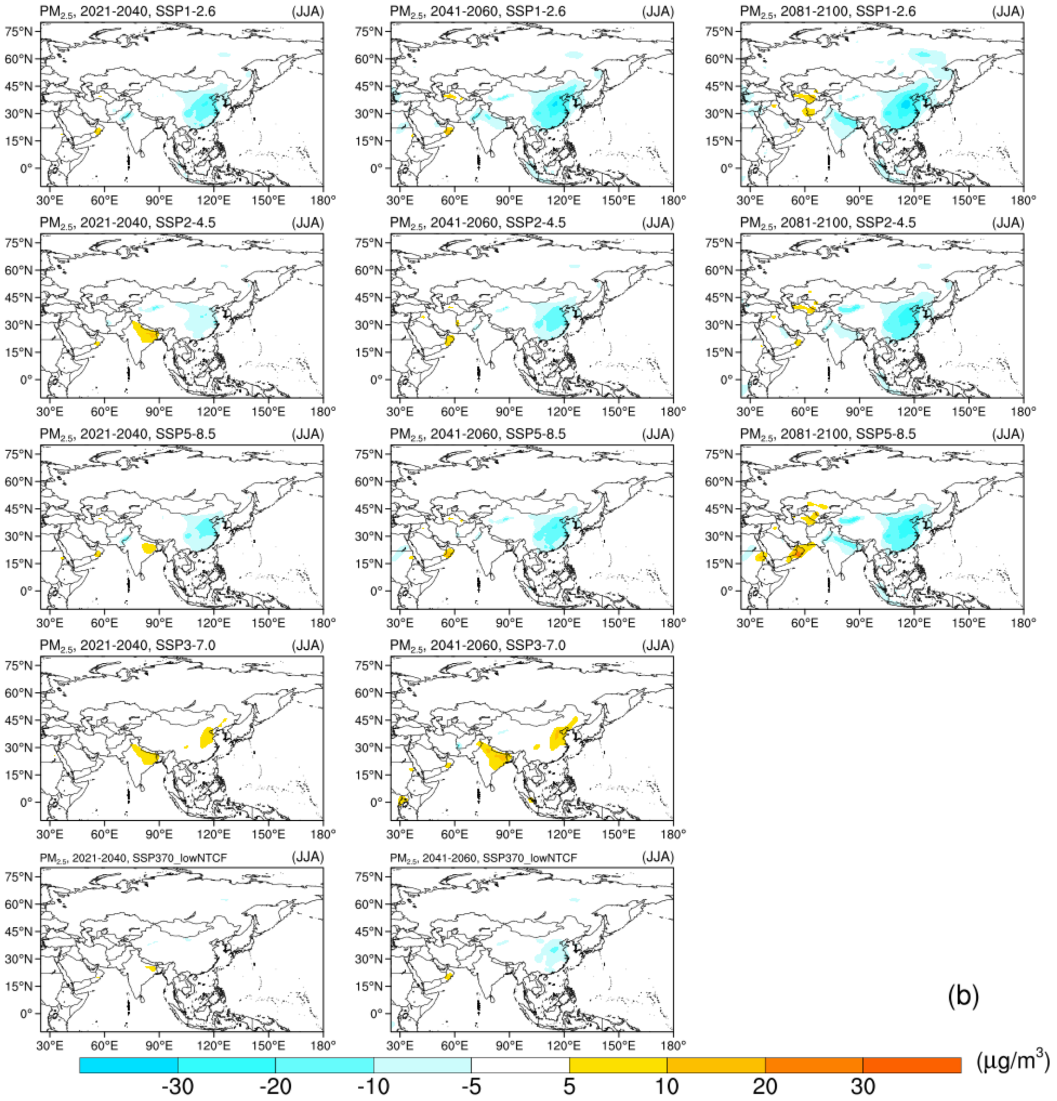
PM2.5, 2021-2040, SSP1-2.6 (JJA)
PM2.5, 2041-2060, SSP1-2.6 (JJA)
PM2.5, 2081-2100, SSP1-2.6 (JJA)
PM2.5, 2021-2040, SSP2-4.5 (JJA)
PM2.5, 2041-2060, SSP2-4.5 (JJA)
PM2.5, 2081-2100, SSP2-4.5 (JJA)
PM2.5, 2021-2040, SSP5-8.5 (JJA)
PM2.5, 2041-2060, SSP5-8.5 (JJA)
PM2.5, 2081-2100, SSP5-8.5 (JJA)
PM2.5, 2021-2040, SSP3-7.0 (JJA)
PM2.5, 2041-2060, SSP3-7.0 (JJA)
PM2.5, 2021-2040, SSP370_lowNTCF (JJA)
PM2.5, 2041-2060, SSP370_lowNTCF (JJA)
75°N
60°N
45°N
30°N
15°N
0°
30°E
60°E
90°E
120°E
150°E
180°
(b)
-30
-20
-10
-5
5
10
20
30
(μg/m3)

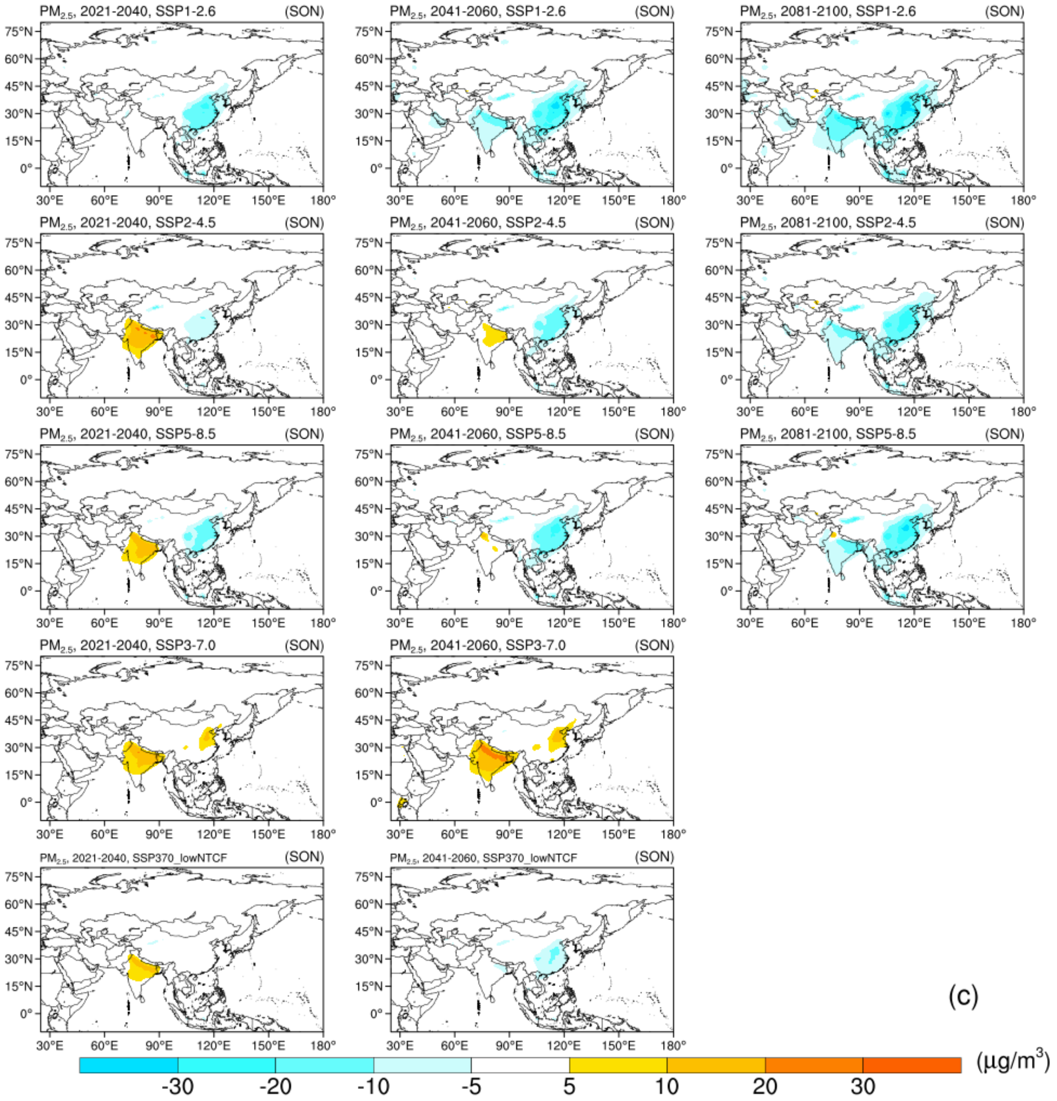
PM2.5, 2021-2040, SSP1-2.6 (SON)
PM2.5, 2041-2060, SSP1-2.6 (SON)
PM2.5, 2081-2100, SSP1-2.6 (SON)
PM2.5, 2021-2040, SSP2-4.5 (SON)
PM2.5, 2041-2060, SSP2-4.5 (SON)
PM2.5, 2081-2100, SSP2-4.5 (SON)
PM2.5, 2021-2040, SSP5-8.5 (SON)
PM2.5, 2041-2060, SSP5-8.5 (SON)
PM2.5, 2081-2100, SSP5-8.5 (SON)
PM2.5, 2021-2040, SSP3-7.0 (SON)
PM2.5, 2041-2060, SSP3-7.0 (SON)
PM2.5, 2021-2040, SSP370_lowNTCF (SON)
PM2.5, 2041-2060, SSP370_lowNTCF (SON)
75°N 60°N 45°N 30°N 15°N 0°
30°E 60°E 90°E 120°E 150°E 180°
(c)
-30 -20 -10 -5 5 10 20 30
(μg/m3)

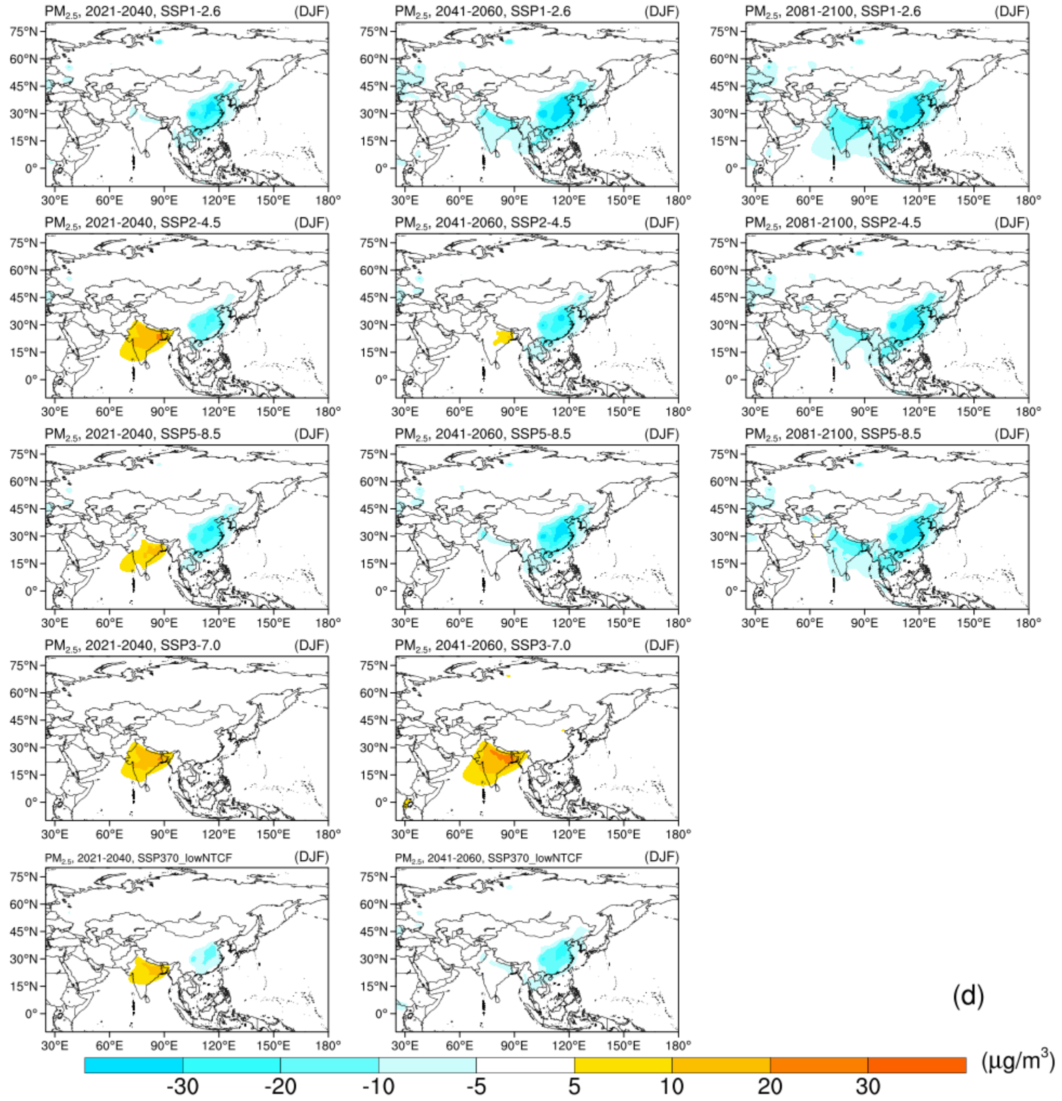

Figure S9 Spatial distribution of projected changes in surface $PM_{2.5}$ concentrations in spring (a), summer (b), autumn (c) and winter (d) (units: $\mu g/m^3$) during 2021–2040, 2041–2060 and 2081–2100 under SSPs, relative to 1995–2014.

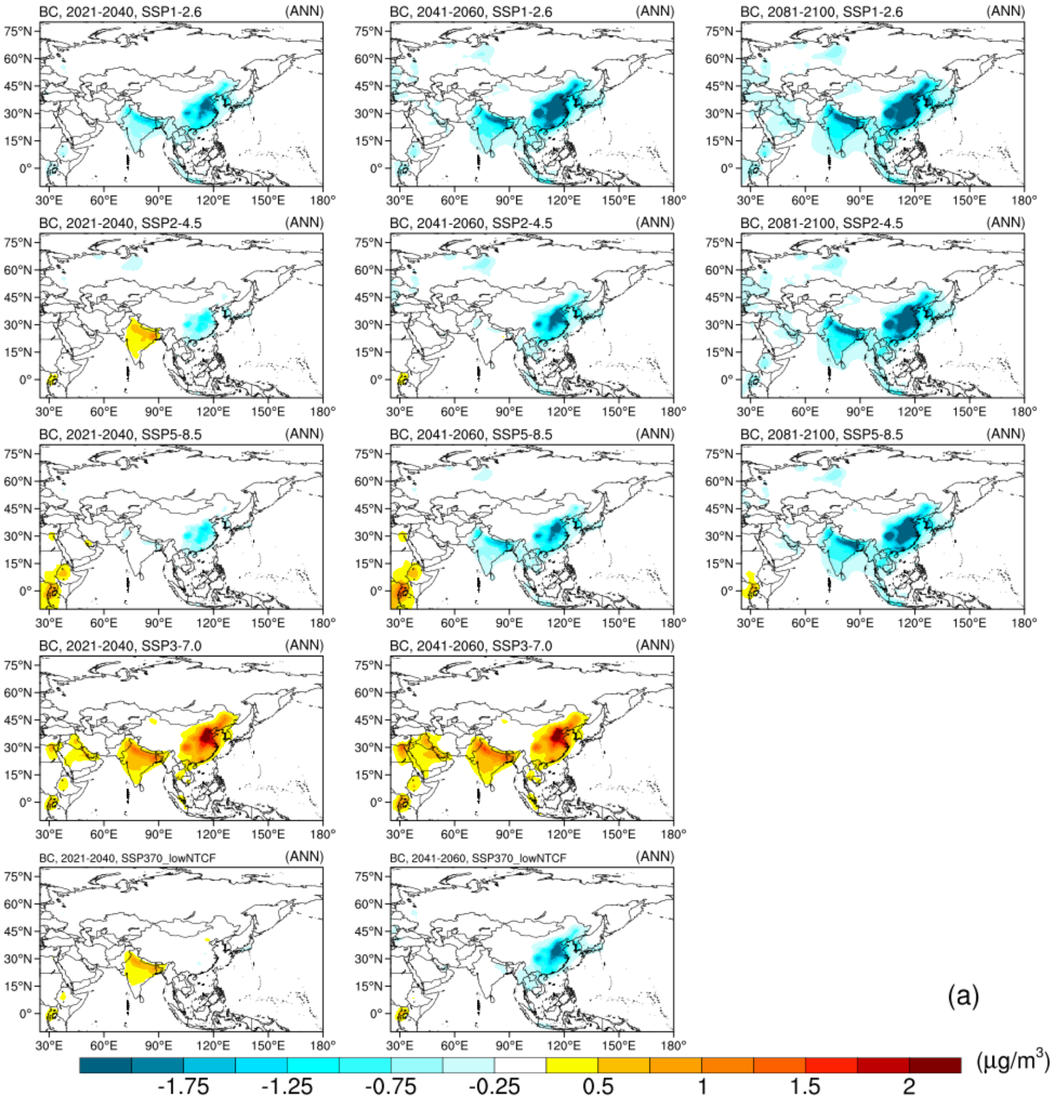
BC, 2021-2040, SSP1-2.6 (ANN)
BC, 2041-2060, SSP1-2.6 (ANN)
BC, 2081-2100, SSP1-2.6 (ANN)
BC, 2021-2040, SSP2-4.5 (ANN)
BC, 2041-2060, SSP2-4.5 (ANN)
BC, 2081-2100, SSP2-4.5 (ANN)
BC, 2021-2040, SSP5-8.5 (ANN)
BC, 2041-2060, SSP5-8.5 (ANN)
BC, 2081-2100, SSP5-8.5 (ANN)
BC, 2021-2040, SSP3-7.0 (ANN)
BC, 2041-2060, SSP3-7.0 (ANN)
BC, 2021-2040, SSP370_lowNTCF (ANN)
BC, 2041-2060, SSP370_lowNTCF (ANN)
75°N
60°N
45°N
30°N
15°N
0°
30°E
60°E
90°E
120°E
150°E
180°
(a)
-1.75
-1.25
-0.75
-0.25
0.5
1
1.5
2
(μg/m3)

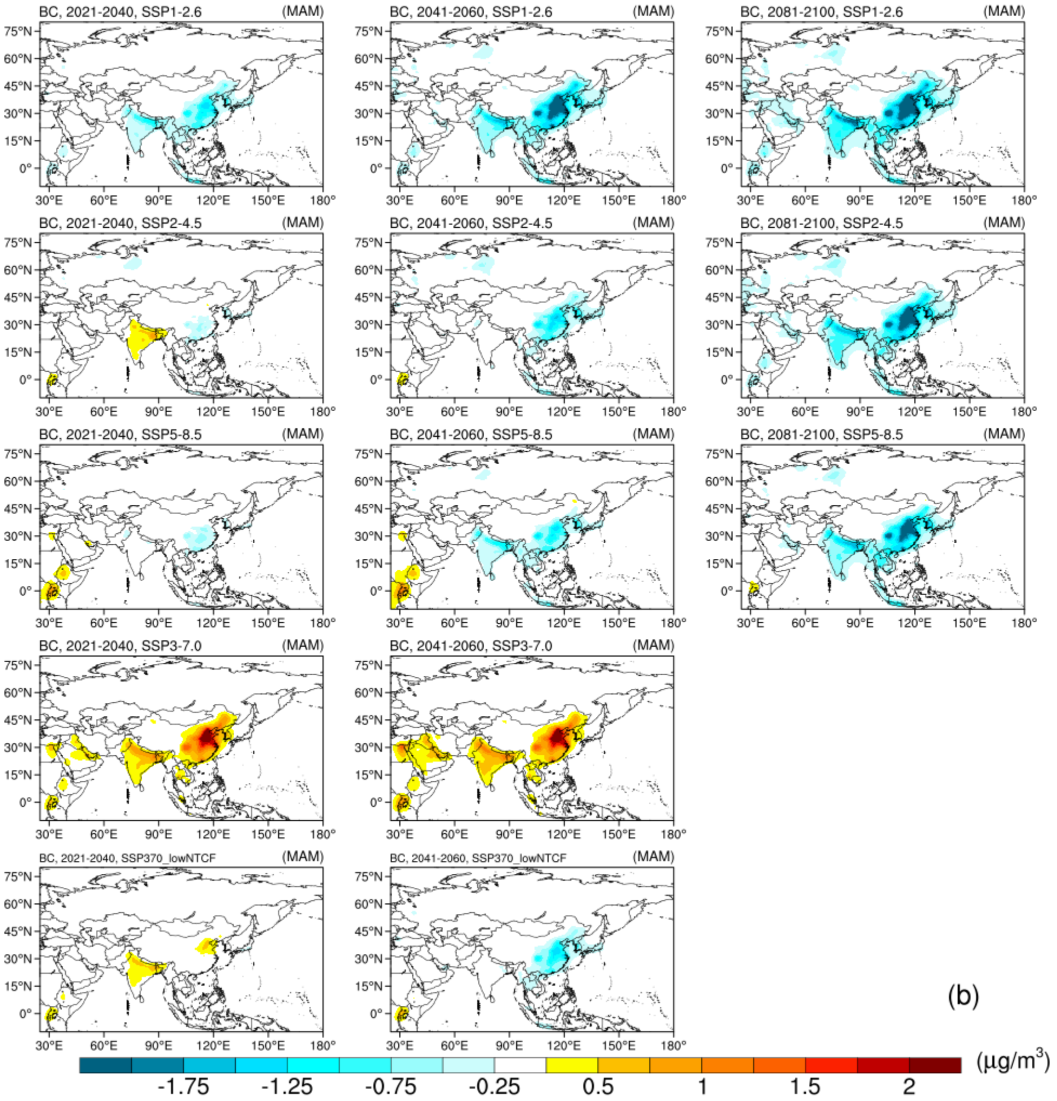

BC, 2021-2040, SSP1-2.6
(MAM)
BC, 2041-2060, SSP1-2.6
(MAM)
BC, 2081-2100, SSP1-2.6
(MAM)
BC, 2021-2040, SSP2-4.5
(MAM)
BC, 2041-2060, SSP2-4.5
(MAM)
BC, 2081-2100, SSP2-4.5
(MAM)
BC, 2021-2040, SSP5-8.5
(MAM)
BC, 2041-2060, SSP5-8.5
(MAM)
BC, 2081-2100, SSP5-8.5
(MAM)
BC, 2021-2040, SSP3-7.0
(MAM)
BC, 2041-2060, SSP3-7.0
(MAM)
BC, 2021-2040, SSP370_lowNTCF
(MAM)
BC, 2041-2060, SSP370_lowNTCF
(MAM)
75°N
60°N
45°N
30°N
15°N
0°
30°E
60°E
90°E
120°E
150°E
180°
(b)
-1.75
-1.25
-0.75
-0.25
0.5
1
1.5
2
(μg/m³)

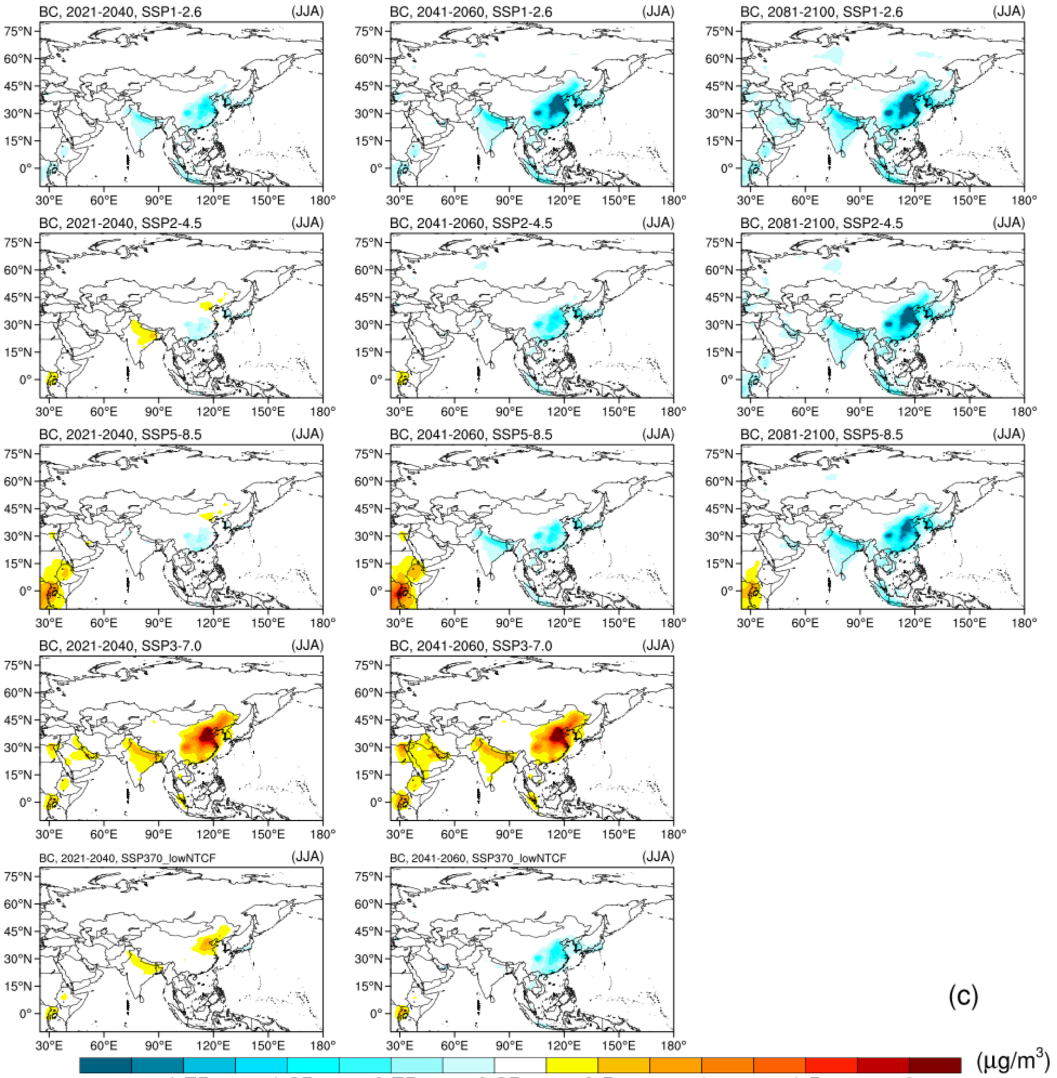
BC, 2021-2040, SSP1-2.6 (JJA)
BC, 2041-2060, SSP1-2.6 (JJA)
BC, 2081-2100, SSP1-2.6 (JJA)
BC, 2021-2040, SSP2-4.5 (JJA)
BC, 2041-2060, SSP2-4.5 (JJA)
BC, 2081-2100, SSP2-4.5 (JJA)
BC, 2021-2040, SSP5-8.5 (JJA)
BC, 2041-2060, SSP5-8.5 (JJA)
BC, 2081-2100, SSP5-8.5 (JJA)
BC, 2021-2040, SSP3-7.0 (JJA)
BC, 2041-2060, SSP3-7.0 (JJA)
BC, 2021-2040, SSP370_lowNTCF (JJA)
BC, 2041-2060, SSP370_lowNTCF (JJA)
75°N 60°N 45°N 30°N 15°N 0°
30°E 60°E 90°E 120°E 150°E 180°
-1.75 -1.25 -0.75 -0.25 0.5 1 1.5 2
(μg/m3)

(c)

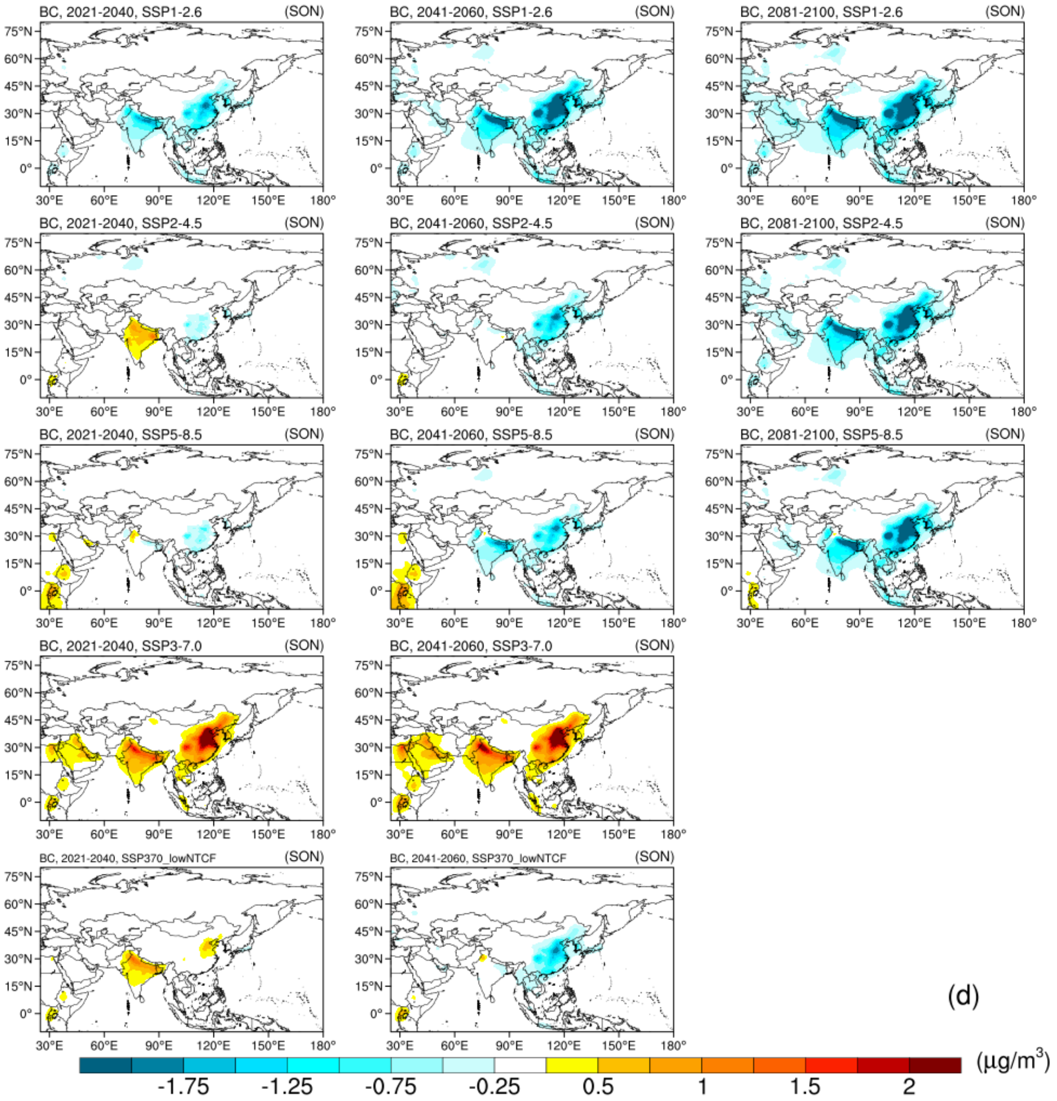

BC, 2021-2040, SSP1-2.6 (SON)
BC, 2041-2060, SSP1-2.6 (SON)
BC, 2081-2100, SSP1-2.6 (SON)
BC, 2021-2040, SSP2-4.5 (SON)
BC, 2041-2060, SSP2-4.5 (SON)
BC, 2081-2100, SSP2-4.5 (SON)
BC, 2021-2040, SSP5-8.5 (SON)
BC, 2041-2060, SSP5-8.5 (SON)
BC, 2081-2100, SSP5-8.5 (SON)
BC, 2021-2040, SSP3-7.0 (SON)
BC, 2041-2060, SSP3-7.0 (SON)
BC, 2021-2040, SSP370_lowNTCF (SON)
BC, 2041-2060, SSP370_lowNTCF (SON)
75°N 60°N 45°N 30°N 15°N 0°
30°E 60°E 90°E 120°E 150°E 180°
(d)
-1.75 -1.25 -0.75 -0.25 0.5 1 1.5 2
(μg/m3)

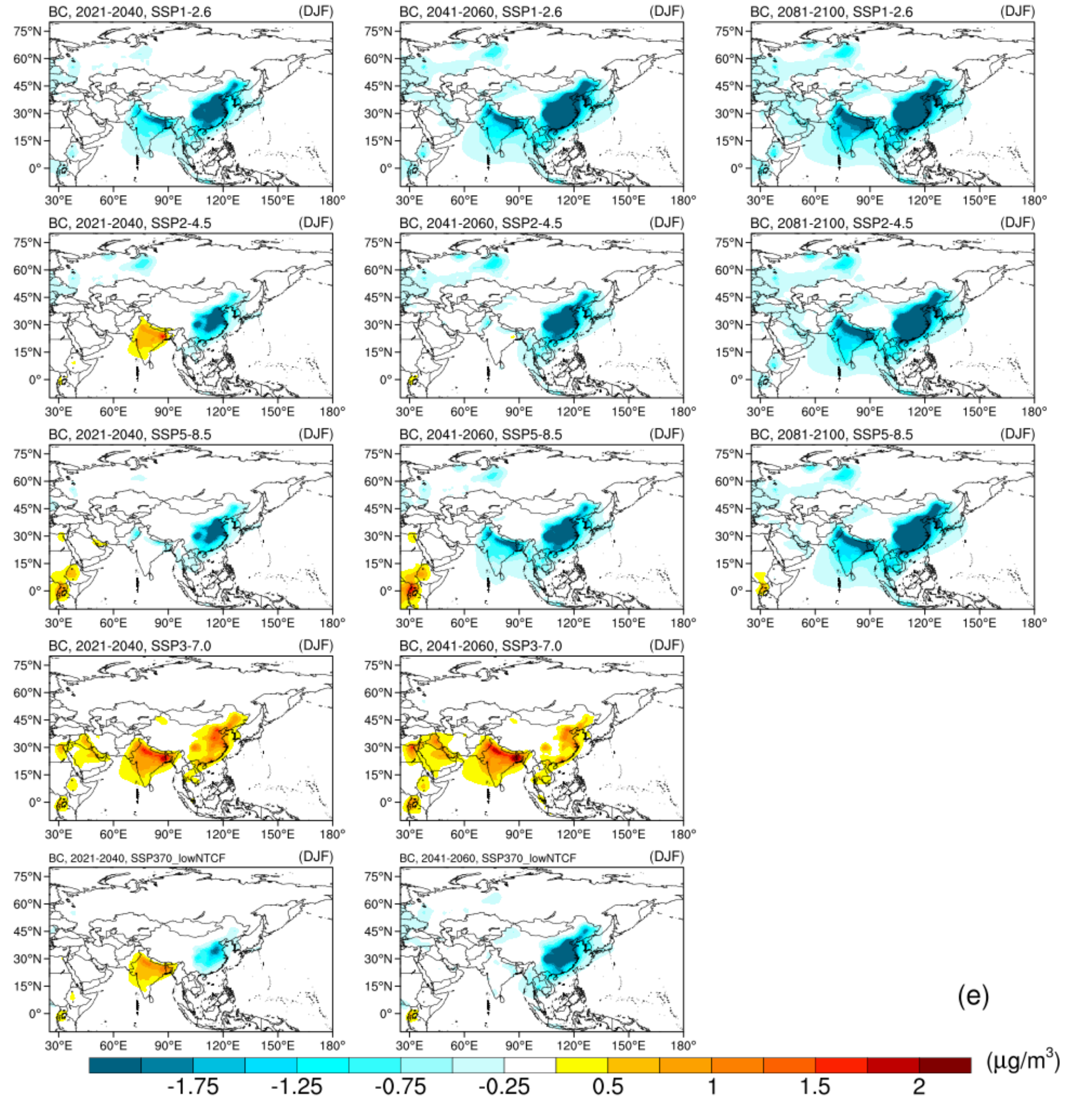

Figure S10 Spatial distribution of projected changes in surface BC concentrations in annual (a), spring (b), summer (c), autumn (d) and winter (e) (units: $\mu g/m^3$) during 2021–2040, 2041–2060 and 2081–2100 under SSPs, relative to 1995–2014.

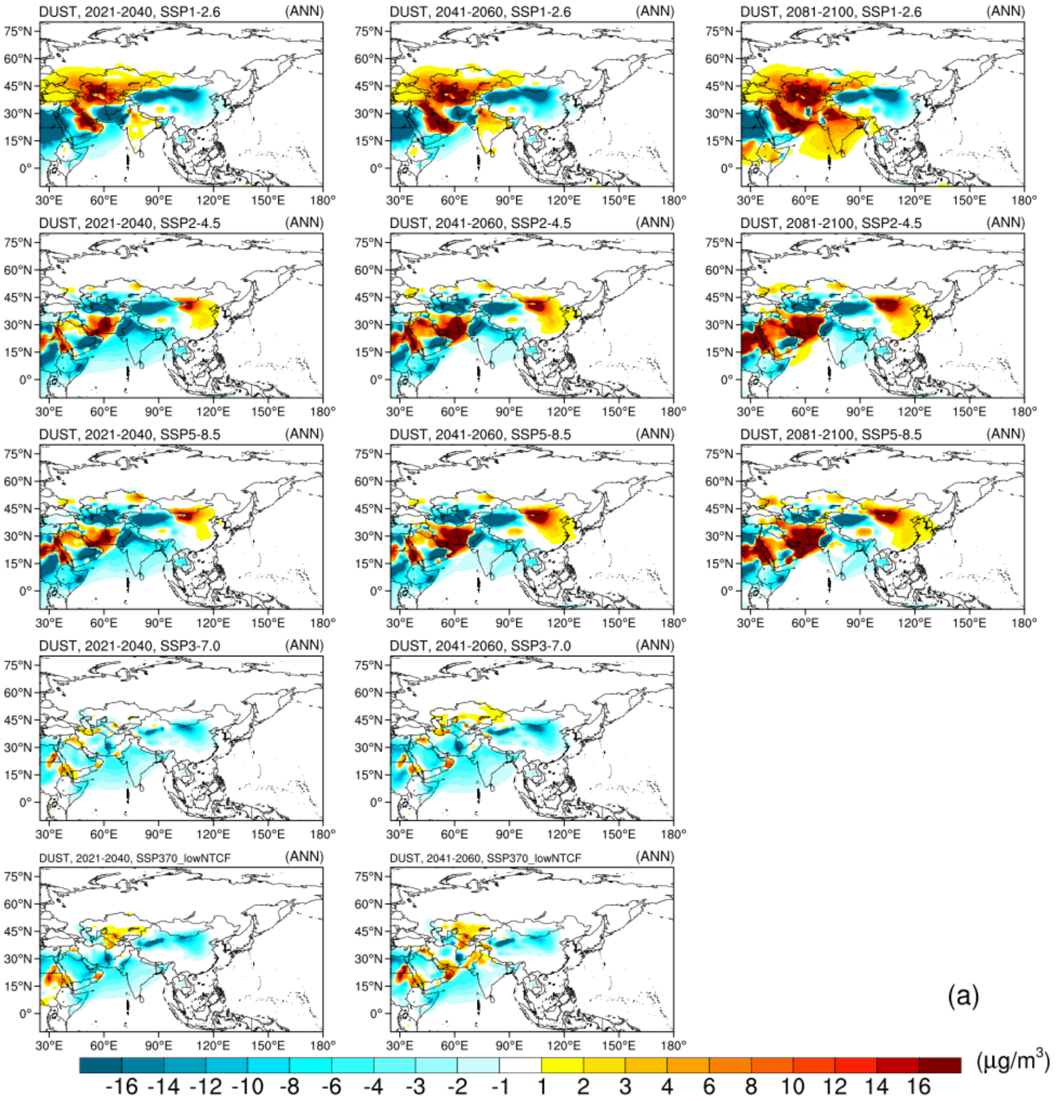
DUST, 2021-2040, SSP1-2.6
(ANN)
DUST, 2041-2060, SSP1-2.6
(ANN)
DUST, 2081-2100, SSP1-2.6
(ANN)
DUST, 2021-2040, SSP2-4.5
(ANN)
DUST, 2041-2060, SSP2-4.5
(ANN)
DUST, 2081-2100, SSP2-4.5
(ANN)
DUST, 2021-2040, SSP5-8.5
(ANN)
DUST, 2041-2060, SSP5-8.5
(ANN)
DUST, 2081-2100, SSP5-8.5
(ANN)
DUST, 2021-2040, SSP3-7.0
(ANN)
DUST, 2041-2060, SSP3-7.0
(ANN)
DUST, 2021-2040, SSP370_lowNTCF
(ANN)
DUST, 2041-2060, SSP370_lowNTCF
(ANN)
75°N
60°N
45°N
30°N
15°N
0°
30°E
60°E
90°E
120°E
150°E
180°
(a)
-16 -14 -12 -10 -8 -6 -4 -3 -2 -1 1 2 3 4 6 8 10 12 14 16
(μg/m³)

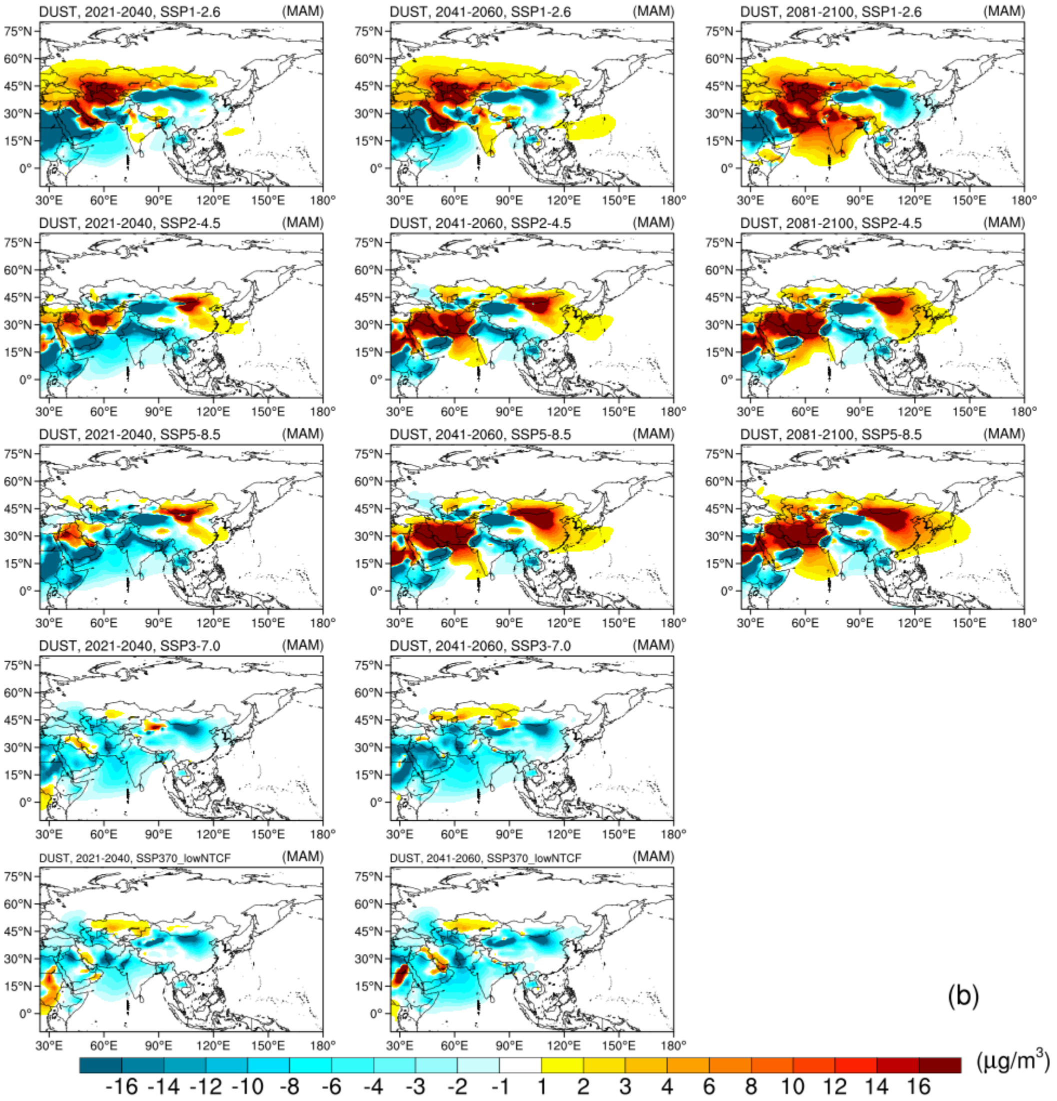

DUST, 2021-2040, SSP1-2.6 (MAM)
DUST, 2041-2060, SSP1-2.6 (MAM)
DUST, 2081-2100, SSP1-2.6 (MAM)
DUST, 2021-2040, SSP2-4.5 (MAM)
DUST, 2041-2060, SSP2-4.5 (MAM)
DUST, 2081-2100, SSP2-4.5 (MAM)
DUST, 2021-2040, SSP5-8.5 (MAM)
DUST, 2041-2060, SSP5-8.5 (MAM)
DUST, 2081-2100, SSP5-8.5 (MAM)
DUST, 2021-2040, SSP3-7.0 (MAM)
DUST, 2041-2060, SSP3-7.0 (MAM)
DUST, 2021-2040, SSP370_lowNTCF (MAM)
DUST, 2041-2060, SSP370_lowNTCF (MAM)
75°N 60°N 45°N 30°N 15°N 0°
30°E 60°E 90°E 120°E 150°E 180°
(b)
-16 -14 -12 -10 -8 -6 -4 -3 -2 -1 1 2 3 4 6 8 10 12 14 16
(μg/m3)

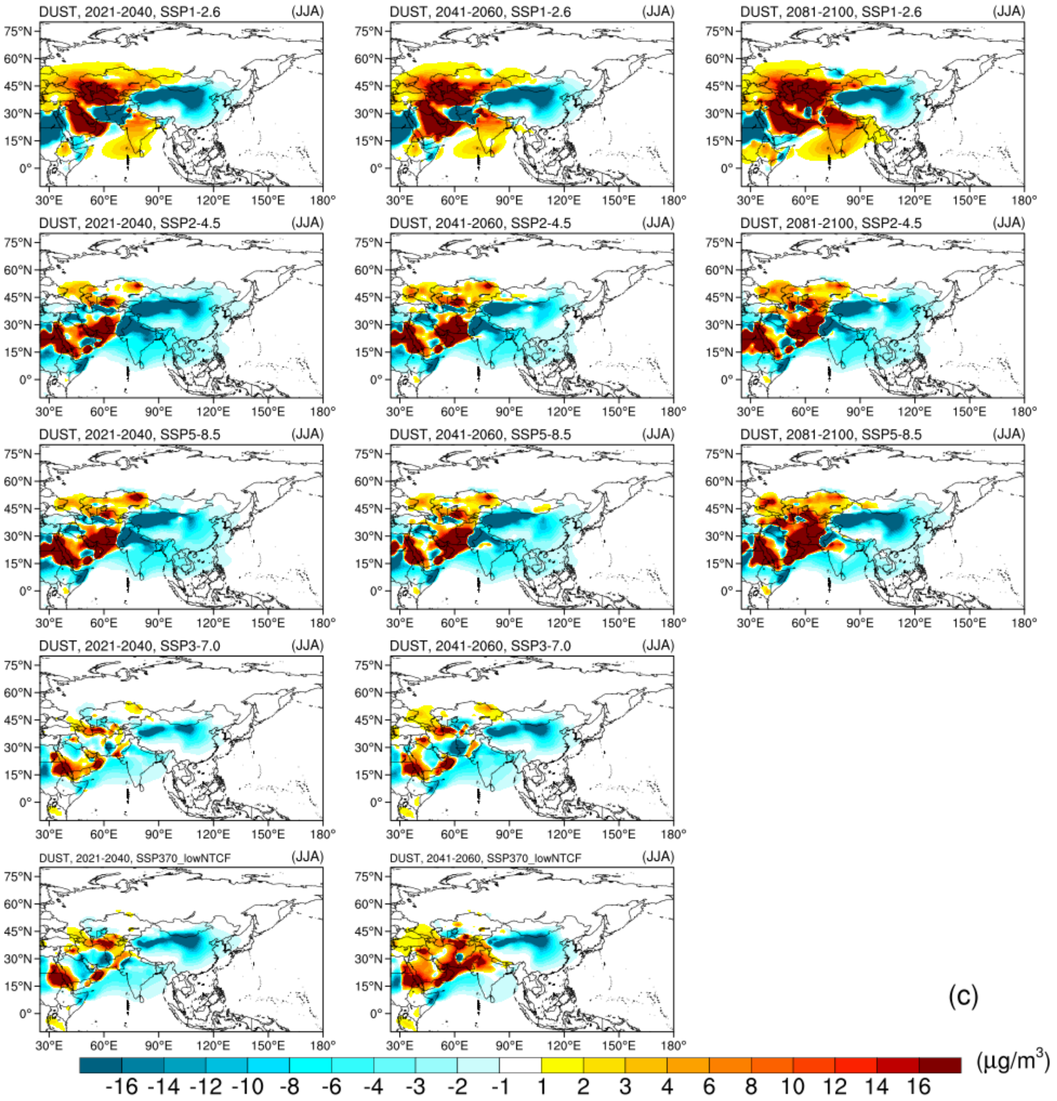
DUST, 2021-2040, SSP1-2.6 (JJA)
DUST, 2041-2060, SSP1-2.6 (JJA)
DUST, 2081-2100, SSP1-2.6 (JJA)
DUST, 2021-2040, SSP2-4.5 (JJA)
DUST, 2041-2060, SSP2-4.5 (JJA)
DUST, 2081-2100, SSP2-4.5 (JJA)
DUST, 2021-2040, SSP5-8.5 (JJA)
DUST, 2041-2060, SSP5-8.5 (JJA)
DUST, 2081-2100, SSP5-8.5 (JJA)
DUST, 2021-2040, SSP3-7.0 (JJA)
DUST, 2041-2060, SSP3-7.0 (JJA)
DUST, 2021-2040, SSP370_lowNTCF (JJA)
DUST, 2041-2060, SSP370_lowNTCF (JJA)
75°N 60°N 45°N 30°N 15°N 0°
30°E 60°E 90°E 120°E 150°E 180°
(c)
-16 -14 -12 -10 -8 -6 -4 -3 -2 -1 1 2 3 4 6 8 10 12 14 16
(μg/m3)

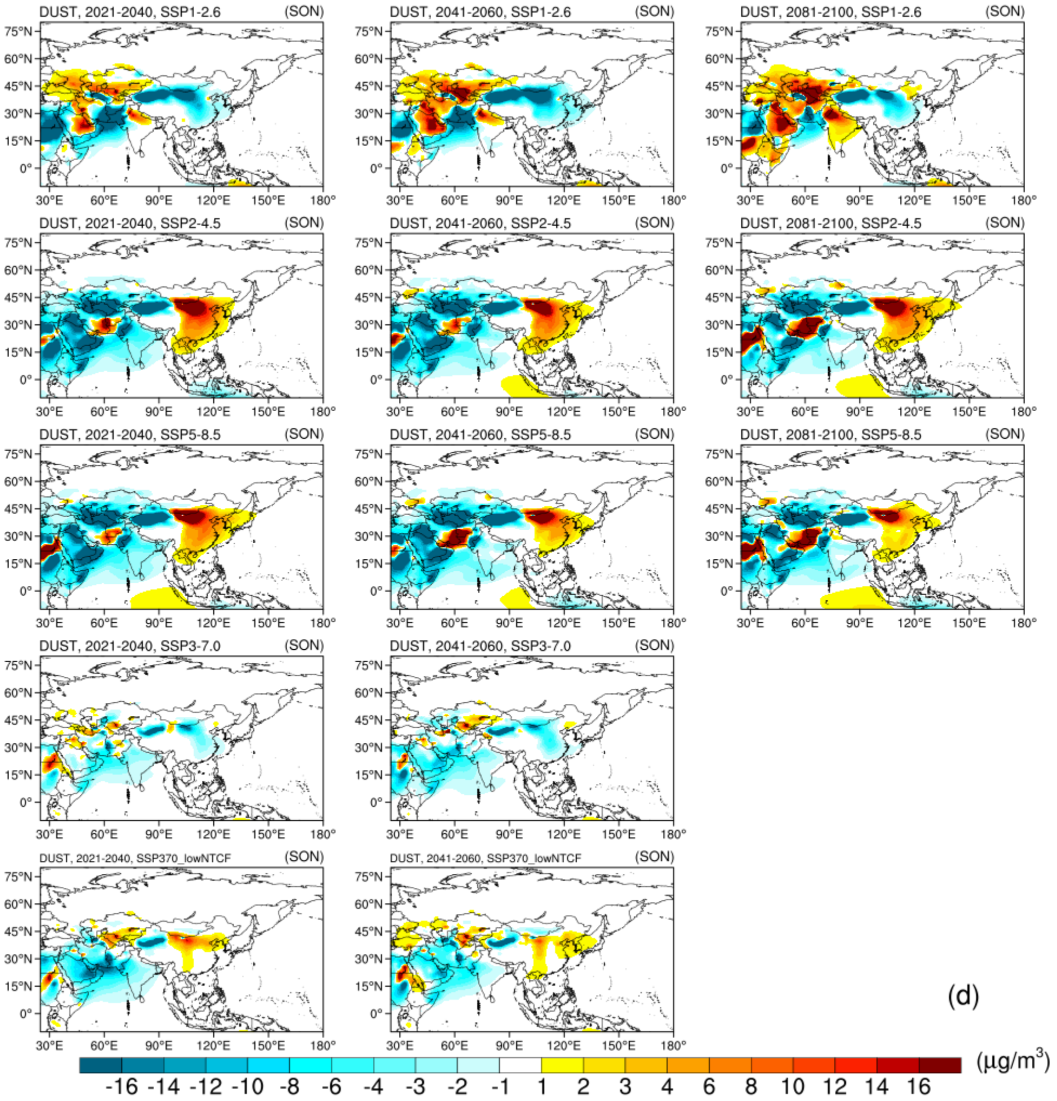
DUST, 2021-2040, SSP1-2.6 (SON)
DUST, 2041-2060, SSP1-2.6 (SON)
DUST, 2081-2100, SSP1-2.6 (SON)
DUST, 2021-2040, SSP2-4.5 (SON)
DUST, 2041-2060, SSP2-4.5 (SON)
DUST, 2081-2100, SSP2-4.5 (SON)
DUST, 2021-2040, SSP5-8.5 (SON)
DUST, 2041-2060, SSP5-8.5 (SON)
DUST, 2081-2100, SSP5-8.5 (SON)
DUST, 2021-2040, SSP3-7.0 (SON)
DUST, 2041-2060, SSP3-7.0 (SON)
DUST, 2021-2040, SSP370_lowNTCF (SON)
DUST, 2041-2060, SSP370_lowNTCF (SON)
75°N 60°N 45°N 30°N 15°N 0°
30°E 60°E 90°E 120°E 150°E 180°
(d)
-16 -14 -12 -10 -8 -6 -4 -3 -2 -1 1 2 3 4 6 8 10 12 14 16
(μg/m3)

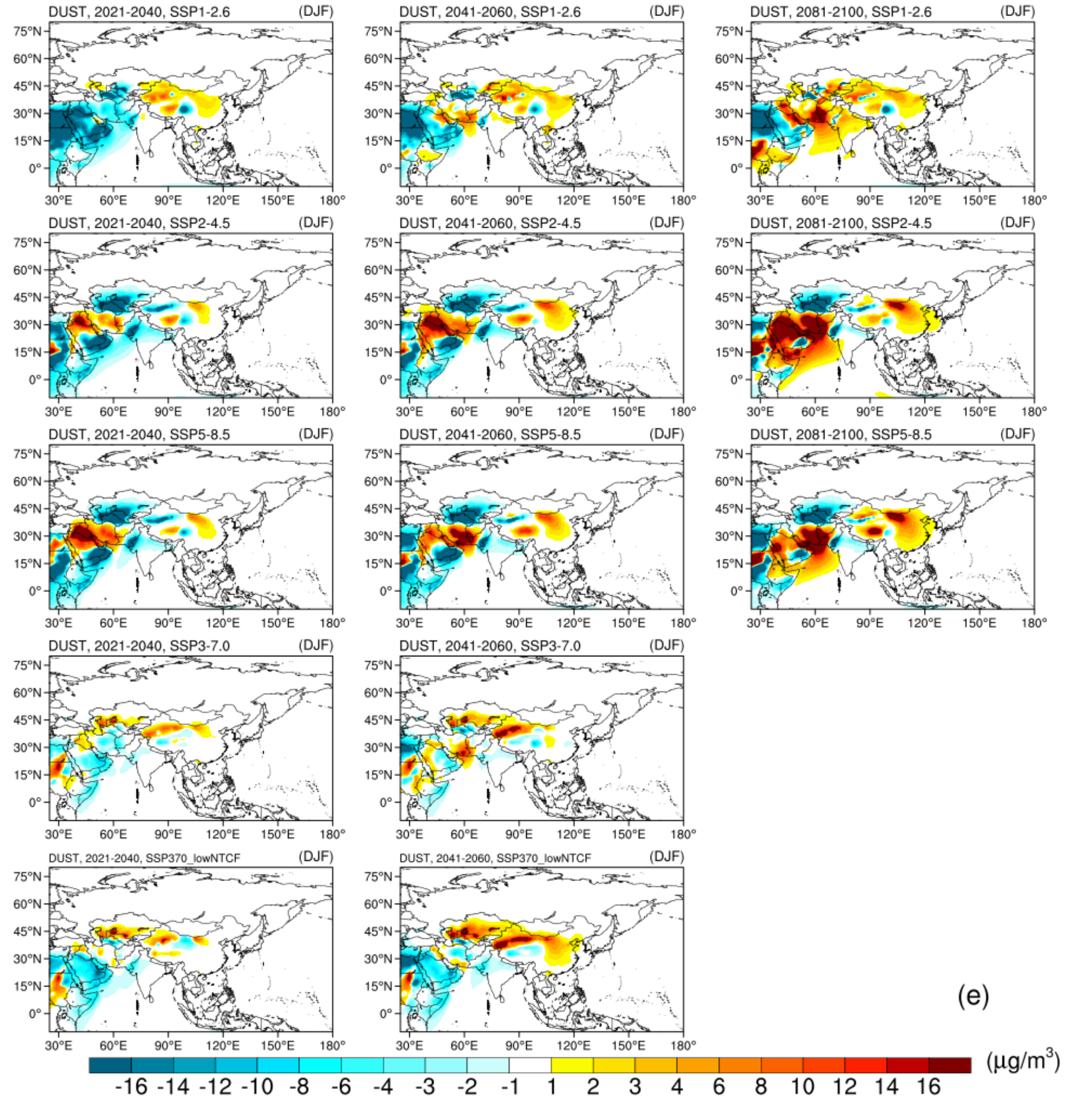

Figure S11 Spatial distribution of projected changes in surface dust concentrations in annual (a), spring (b), summer (c), autumn (d) and winter (e) (units: $\mu g/m^3$) during 2021–2040, 2041–2060 and 2081–2100 under SSPs, relative to 1995–2014.

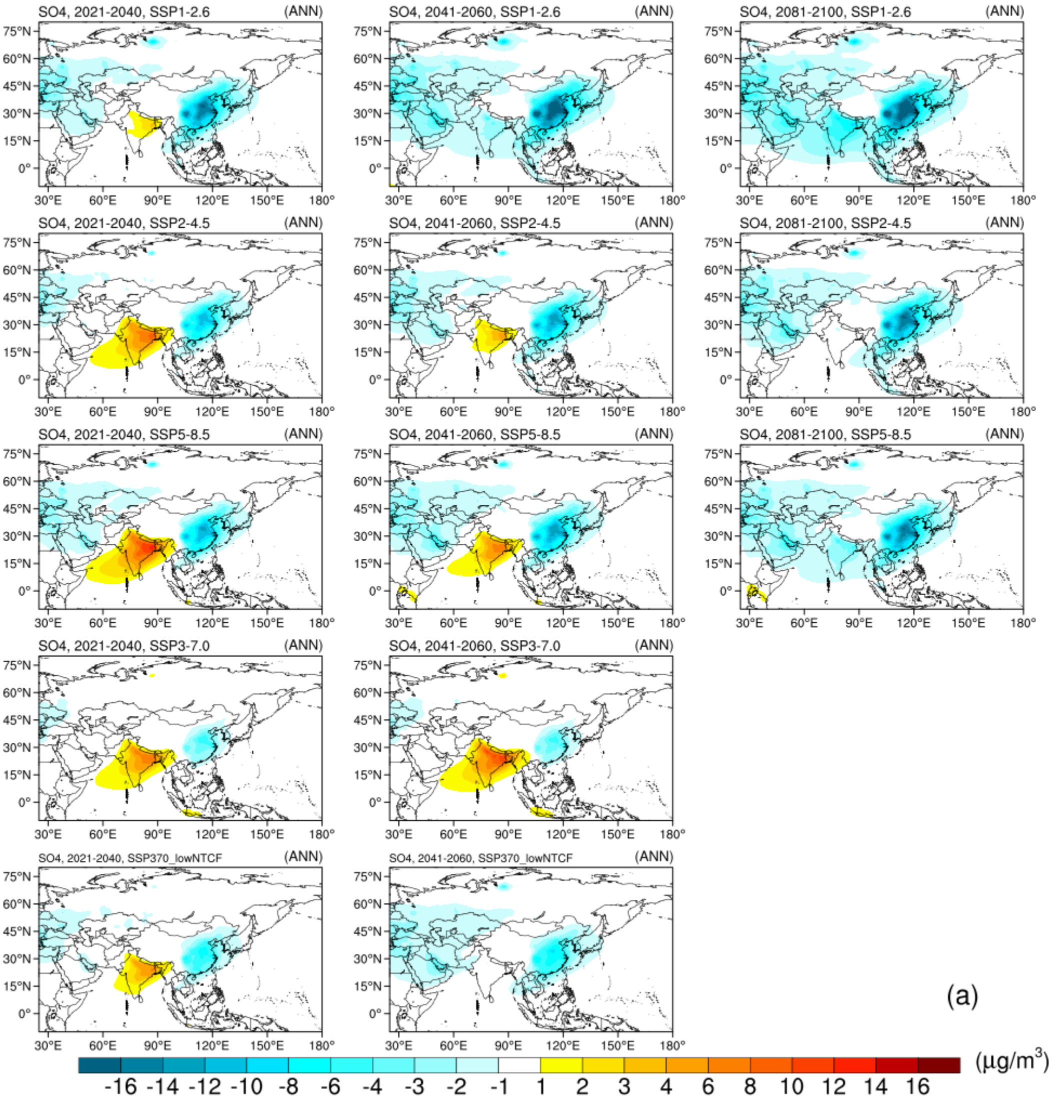
SO4, 2021-2040, SSP1-2.6 (ANN)
SO4, 2041-2060, SSP1-2.6 (ANN)
SO4, 2081-2100, SSP1-2.6 (ANN)
SO4, 2021-2040, SSP2-4.5 (ANN)
SO4, 2041-2060, SSP2-4.5 (ANN)
SO4, 2081-2100, SSP2-4.5 (ANN)
SO4, 2021-2040, SSP5-8.5 (ANN)
SO4, 2041-2060, SSP5-8.5 (ANN)
SO4, 2081-2100, SSP5-8.5 (ANN)
SO4, 2021-2040, SSP3-7.0 (ANN)
SO4, 2041-2060, SSP3-7.0 (ANN)
SO4, 2021-2040, SSP370_lowNTCF (ANN)
SO4, 2041-2060, SSP370_lowNTCF (ANN)
75°N 60°N 45°N 30°N 15°N 0°
30°E 60°E 90°E 120°E 150°E 180°
(a)
-16 -14 -12 -10 -8 -6 -4 -3 -2 -1 1 2 3 4 6 8 10 12 14 16
(μg/m3)

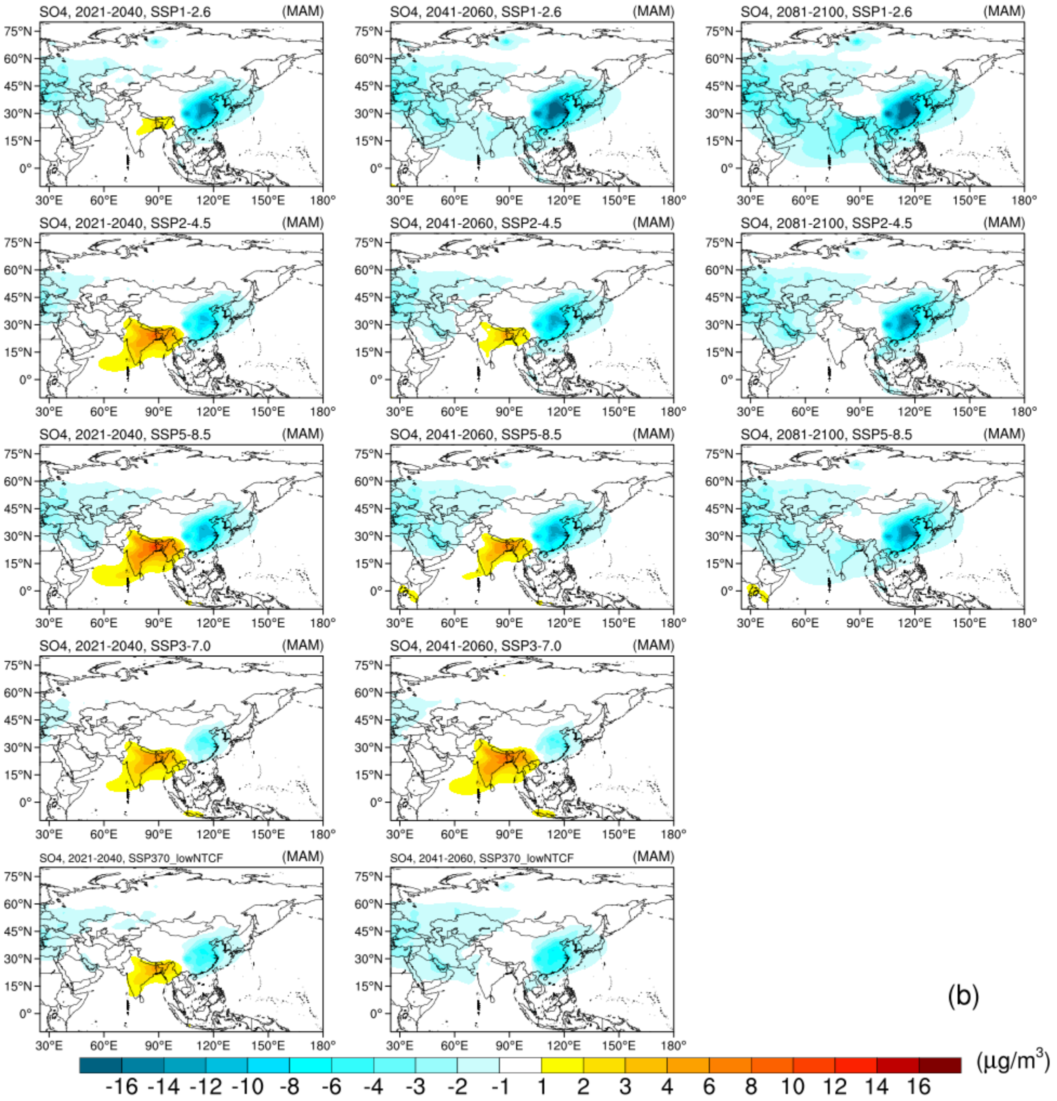

SO4, 2021-2040, SSP1-2.6 (MAM)
SO4, 2041-2060, SSP1-2.6 (MAM)
SO4, 2081-2100, SSP1-2.6 (MAM)
SO4, 2021-2040, SSP2-4.5 (MAM)
SO4, 2041-2060, SSP2-4.5 (MAM)
SO4, 2081-2100, SSP2-4.5 (MAM)
SO4, 2021-2040, SSP5-8.5 (MAM)
SO4, 2041-2060, SSP5-8.5 (MAM)
SO4, 2081-2100, SSP5-8.5 (MAM)
SO4, 2021-2040, SSP3-7.0 (MAM)
SO4, 2041-2060, SSP3-7.0 (MAM)
SO4, 2021-2040, SSP370_lowNTCF (MAM)
SO4, 2041-2060, SSP370_lowNTCF (MAM)
75°N 60°N 45°N 30°N 15°N 0°
30°E 60°E 90°E 120°E 150°E 180°
(b)
-16 -14 -12 -10 -8 -6 -4 -3 -2 -1 1 2 3 4 6 8 10 12 14 16
(μg/m3)

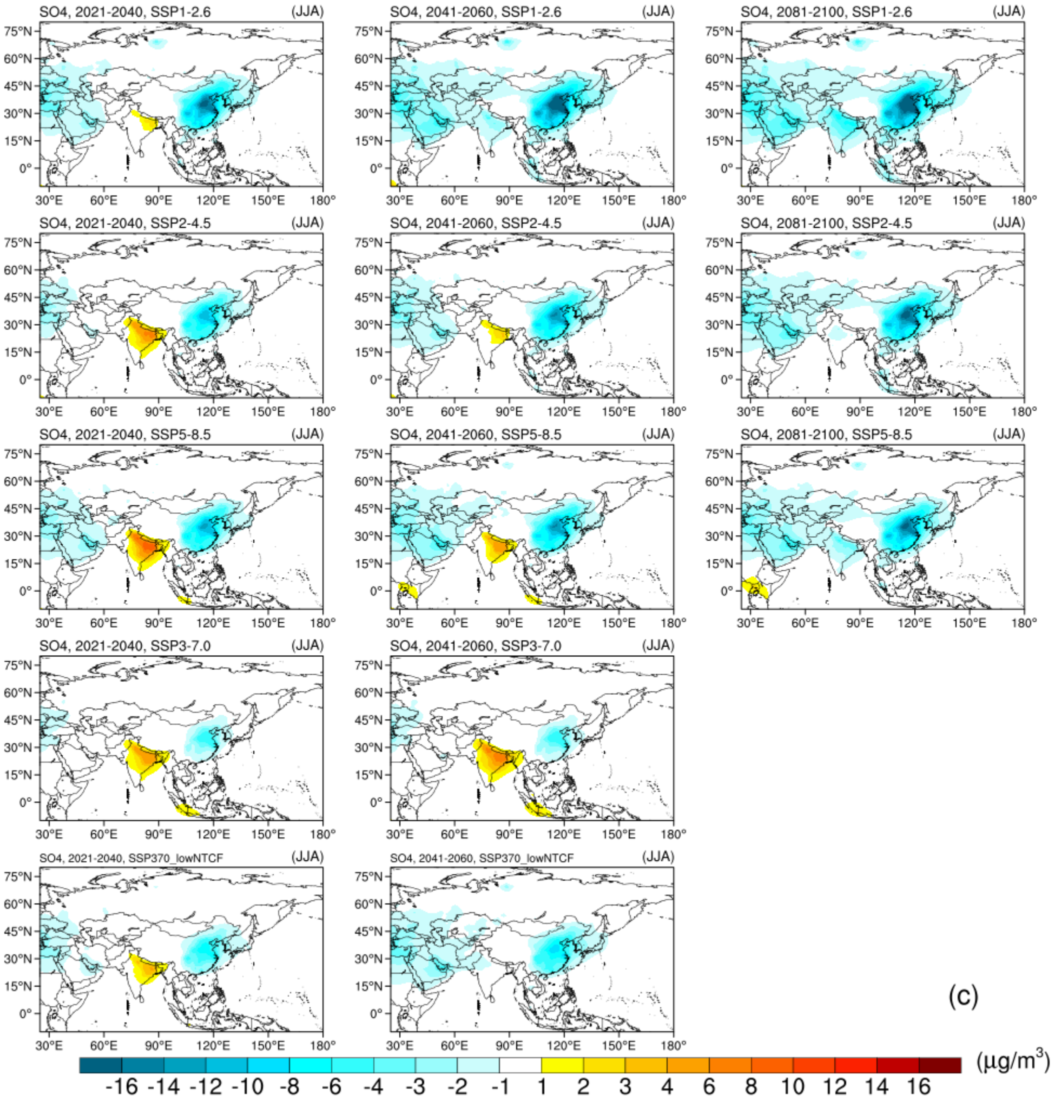
SO4, 2021-2040, SSP1-2.6 (JJA)
SO4, 2041-2060, SSP1-2.6 (JJA)
SO4, 2081-2100, SSP1-2.6 (JJA)
SO4, 2021-2040, SSP2-4.5 (JJA)
SO4, 2041-2060, SSP2-4.5 (JJA)
SO4, 2081-2100, SSP2-4.5 (JJA)
SO4, 2021-2040, SSP5-8.5 (JJA)
SO4, 2041-2060, SSP5-8.5 (JJA)
SO4, 2081-2100, SSP5-8.5 (JJA)
SO4, 2021-2040, SSP3-7.0 (JJA)
SO4, 2041-2060, SSP3-7.0 (JJA)
SO4, 2021-2040, SSP370_lowNTCF (JJA)
SO4, 2041-2060, SSP370_lowNTCF (JJA)
75°N 60°N 45°N 30°N 15°N 0°
30°E 60°E 90°E 120°E 150°E 180°
(c)
-16 -14 -12 -10 -8 -6 -4 -3 -2 -1 1 2 3 4 6 8 10 12 14 16
(μg/m3)

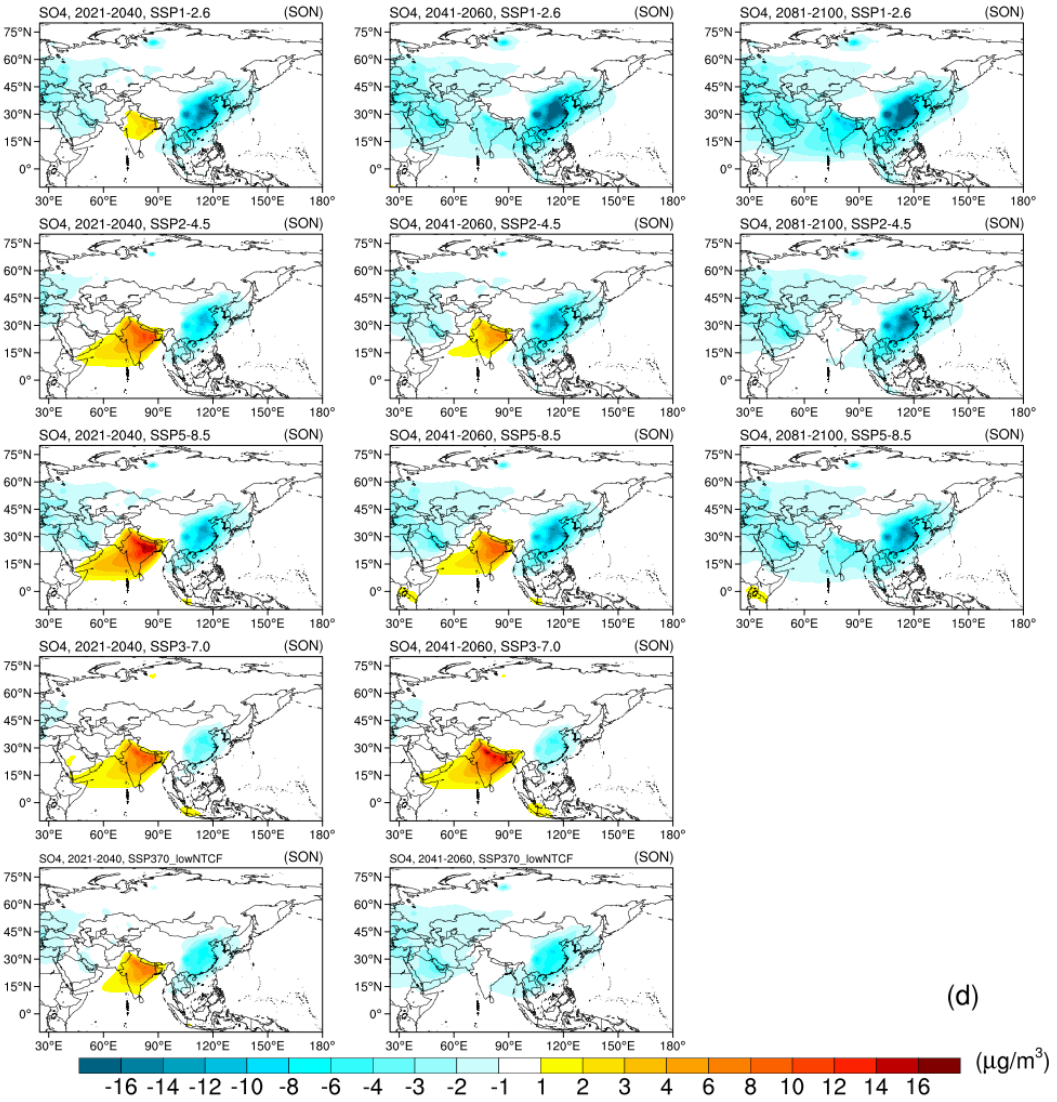
SO4, 2021-2040, SSP1-2.6 (SON)
SO4, 2041-2060, SSP1-2.6 (SON)
SO4, 2081-2100, SSP1-2.6 (SON)
SO4, 2021-2040, SSP2-4.5 (SON)
SO4, 2041-2060, SSP2-4.5 (SON)
SO4, 2081-2100, SSP2-4.5 (SON)
SO4, 2021-2040, SSP5-8.5 (SON)
SO4, 2041-2060, SSP5-8.5 (SON)
SO4, 2081-2100, SSP5-8.5 (SON)
SO4, 2021-2040, SSP3-7.0 (SON)
SO4, 2041-2060, SSP3-7.0 (SON)
SO4, 2021-2040, SSP370_lowNTCF (SON)
SO4, 2041-2060, SSP370_lowNTCF (SON)
75°N
60°N
45°N
30°N
15°N
0°
30°E
60°E
90°E
120°E
150°E
180°
(d)
-16 -14 -12 -10 -8 -6 -4 -3 -2 -1 1 2 3 4 6 8 10 12 14 16
(μg/m3)

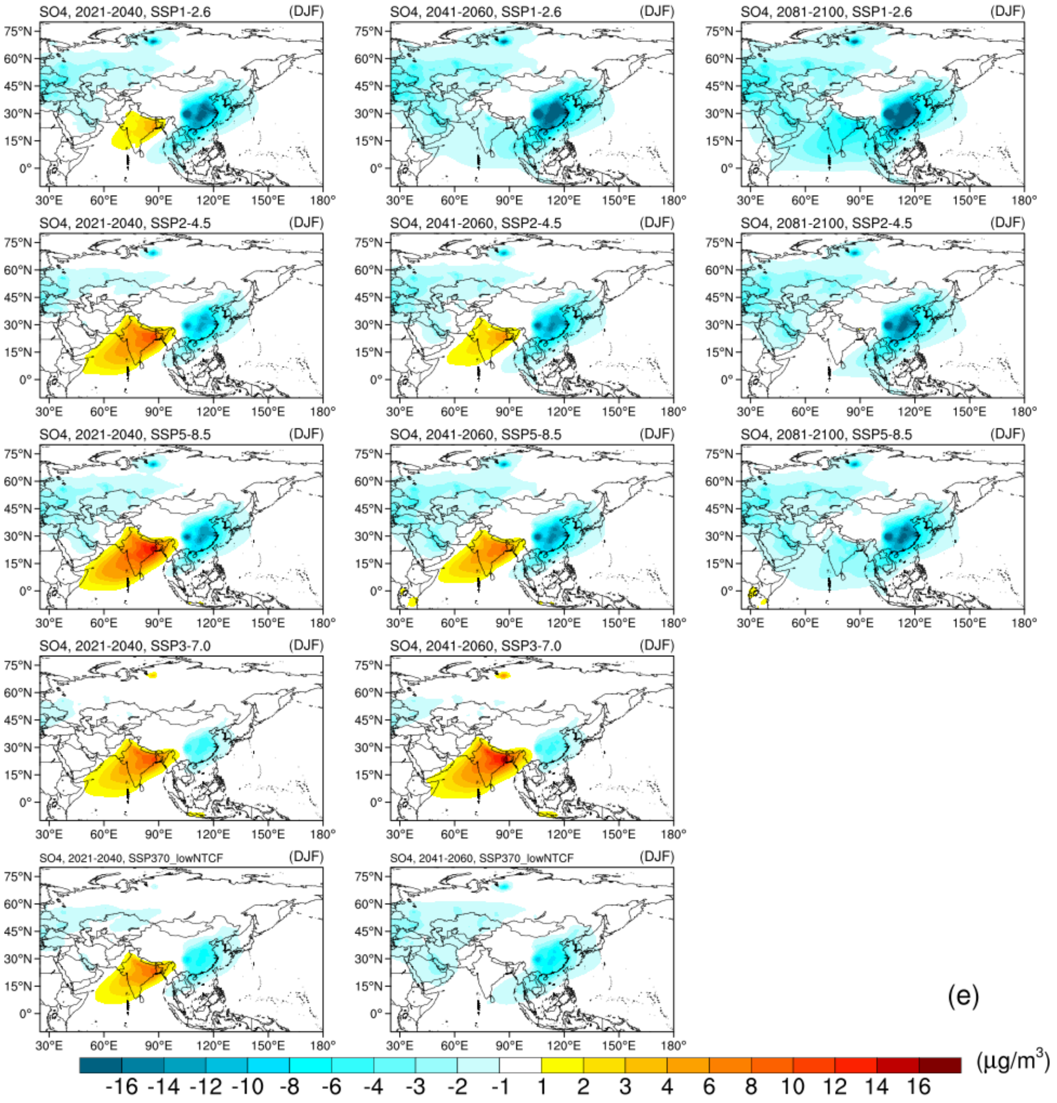

Figure S12 Spatial distribution of projected changes in surface OA concentrations in annual (a), spring (b), summer (c), autumn (d) and winter (e) (units: $\mu g/m^3$) during 2021–2040, 2041–2060 and 2081–2100 under SSPs, relative to 1995–2014.

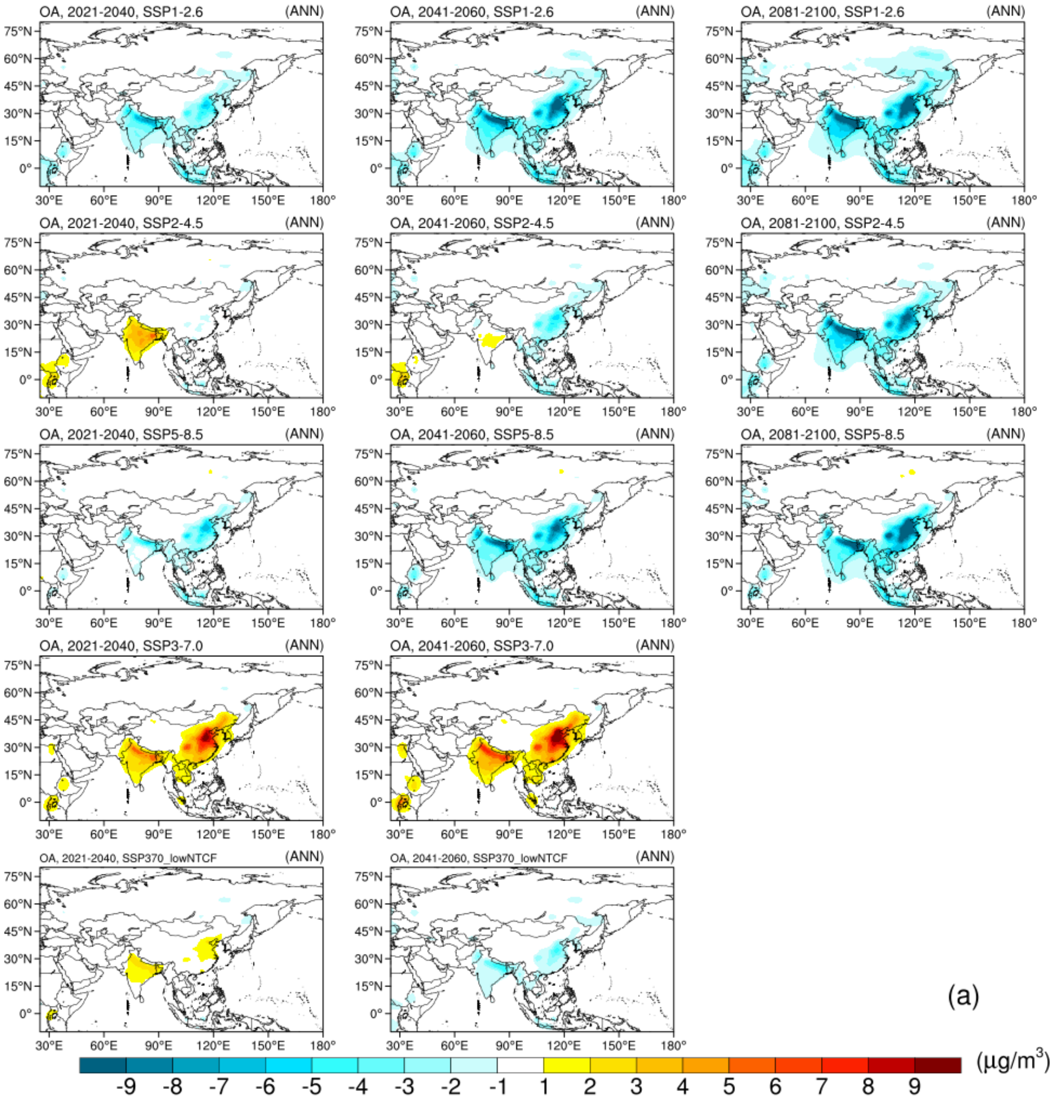
OA, 2021-2040, SSP1-2.6 (ANN)
OA, 2041-2060, SSP1-2.6 (ANN)
OA, 2081-2100, SSP1-2.6 (ANN)
OA, 2021-2040, SSP2-4.5 (ANN)
OA, 2041-2060, SSP2-4.5 (ANN)
OA, 2081-2100, SSP2-4.5 (ANN)
OA, 2021-2040, SSP5-8.5 (ANN)
OA, 2041-2060, SSP5-8.5 (ANN)
OA, 2081-2100, SSP5-8.5 (ANN)
OA, 2021-2040, SSP3-7.0 (ANN)
OA, 2041-2060, SSP3-7.0 (ANN)
OA, 2021-2040, SSP370_lowNTCF (ANN)
OA, 2041-2060, SSP370_lowNTCF (ANN)
75°N 60°N 45°N 30°N 15°N 0°
30°E 60°E 90°E 120°E 150°E 180°
(a)
-9 -8 -7 -6 -5 -4 -3 -2 -1 1 2 3 4 5 6 7 8 9
(μg/m3)

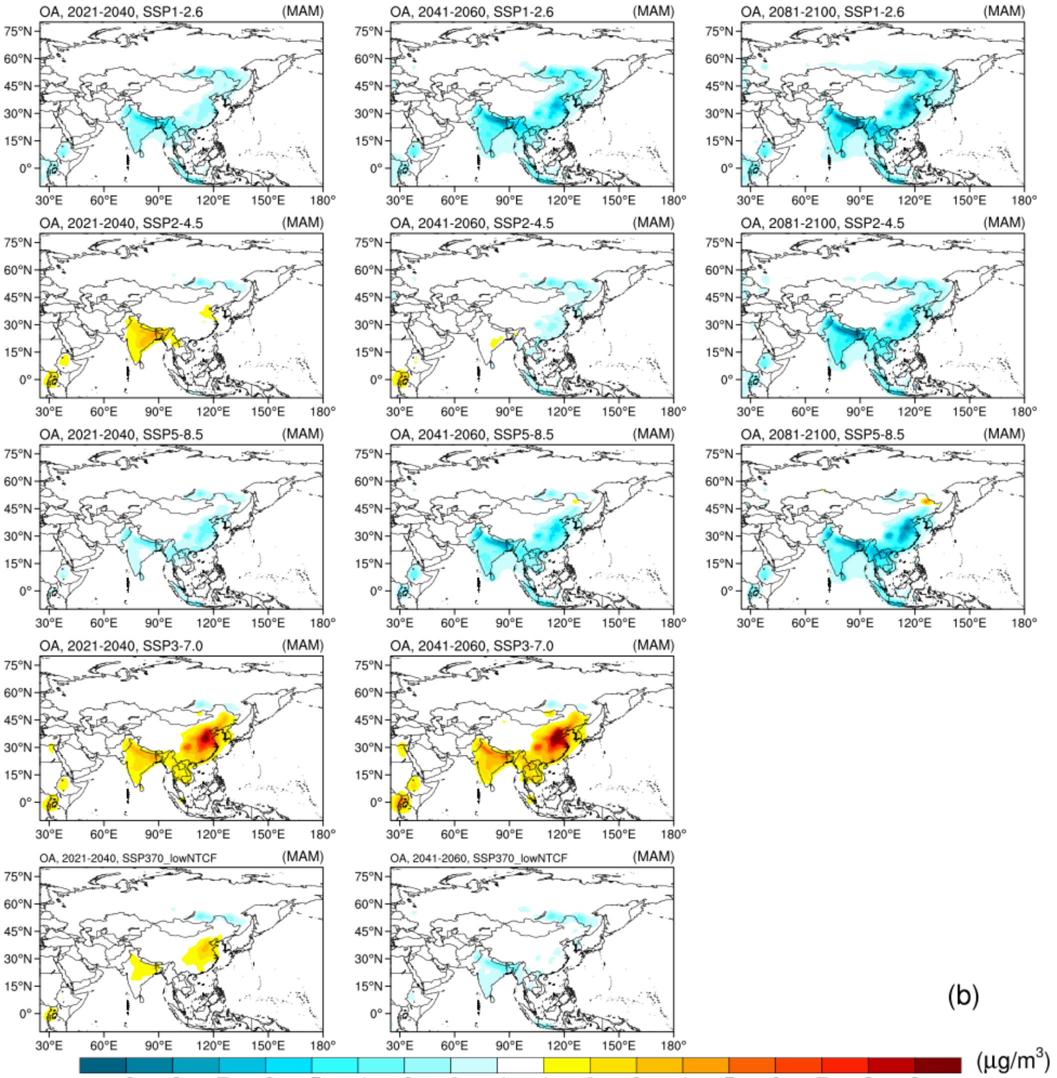
OA, 2021-2040, SSP1-2.6 (MAM)
OA, 2041-2060, SSP1-2.6 (MAM)
OA, 2081-2100, SSP1-2.6 (MAM)
OA, 2021-2040, SSP2-4.5 (MAM)
OA, 2041-2060, SSP2-4.5 (MAM)
OA, 2081-2100, SSP2-4.5 (MAM)
OA, 2021-2040, SSP5-8.5 (MAM)
OA, 2041-2060, SSP5-8.5 (MAM)
OA, 2081-2100, SSP5-8.5 (MAM)
OA, 2021-2040, SSP3-7.0 (MAM)
OA, 2041-2060, SSP3-7.0 (MAM)
OA, 2021-2040, SSP370_lowNTCF (MAM)
OA, 2041-2060, SSP370_lowNTCF (MAM)
75°N 60°N 45°N 30°N 15°N 0°
30°E 60°E 90°E 120°E 150°E 180°
(b)
-9 -8 -7 -6 -5 -4 -3 -2 -1 1 2 3 4 5 6 7 8 9
(μg/m³)

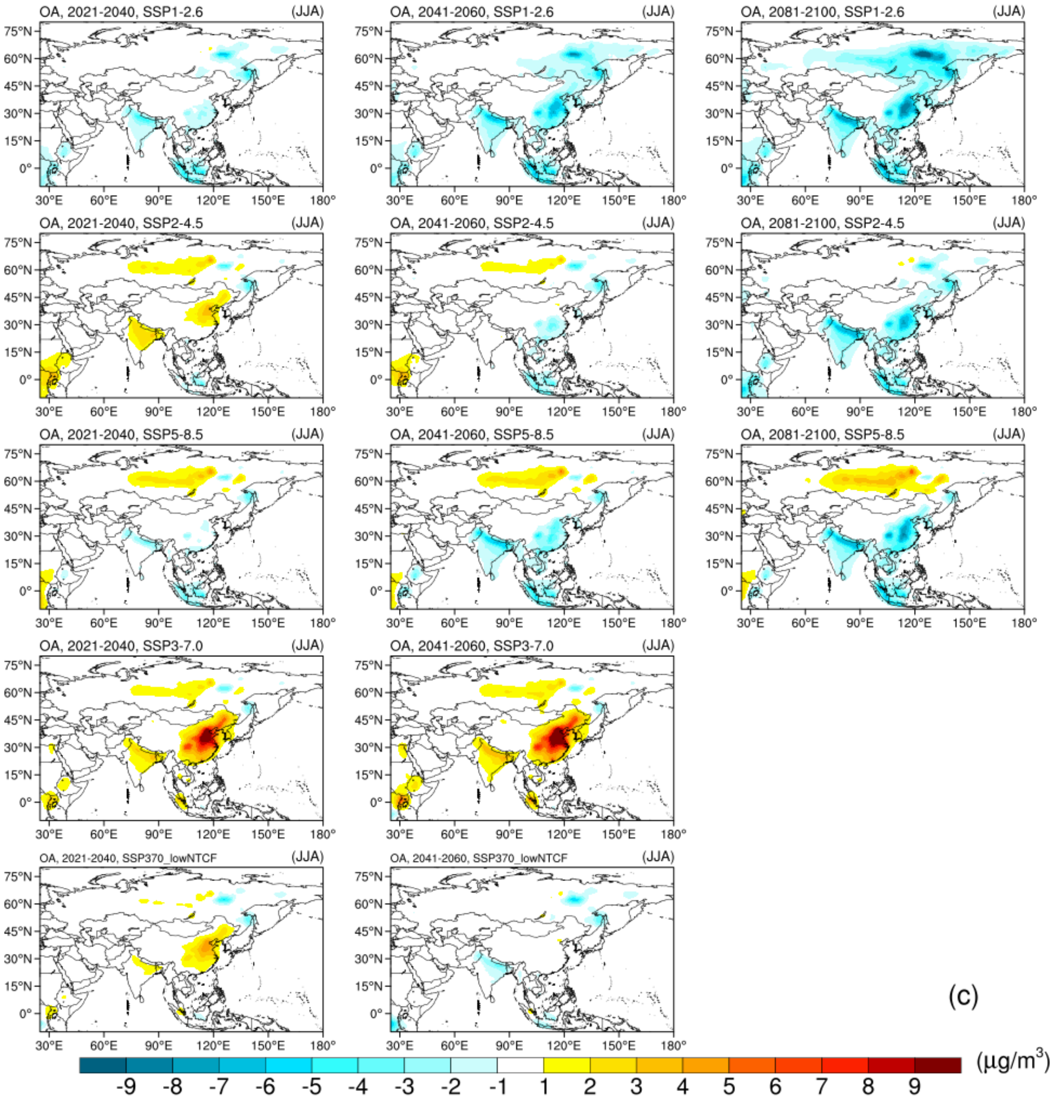

OA, 2021-2040, SSP1-2.6 (JJA)
OA, 2041-2060, SSP1-2.6 (JJA)
OA, 2081-2100, SSP1-2.6 (JJA)
OA, 2021-2040, SSP2-4.5 (JJA)
OA, 2041-2060, SSP2-4.5 (JJA)
OA, 2081-2100, SSP2-4.5 (JJA)
OA, 2021-2040, SSP5-8.5 (JJA)
OA, 2041-2060, SSP5-8.5 (JJA)
OA, 2081-2100, SSP5-8.5 (JJA)
OA, 2021-2040, SSP3-7.0 (JJA)
OA, 2041-2060, SSP3-7.0 (JJA)
OA, 2021-2040, SSP370_lowNTCF (JJA)
OA, 2041-2060, SSP370_lowNTCF (JJA)
75°N 60°N 45°N 30°N 15°N 0°
30°E 60°E 90°E 120°E 150°E 180°
(c)
-9 -8 -7 -6 -5 -4 -3 -2 -1 1 2 3 4 5 6 7 8 9
(μg/m3)

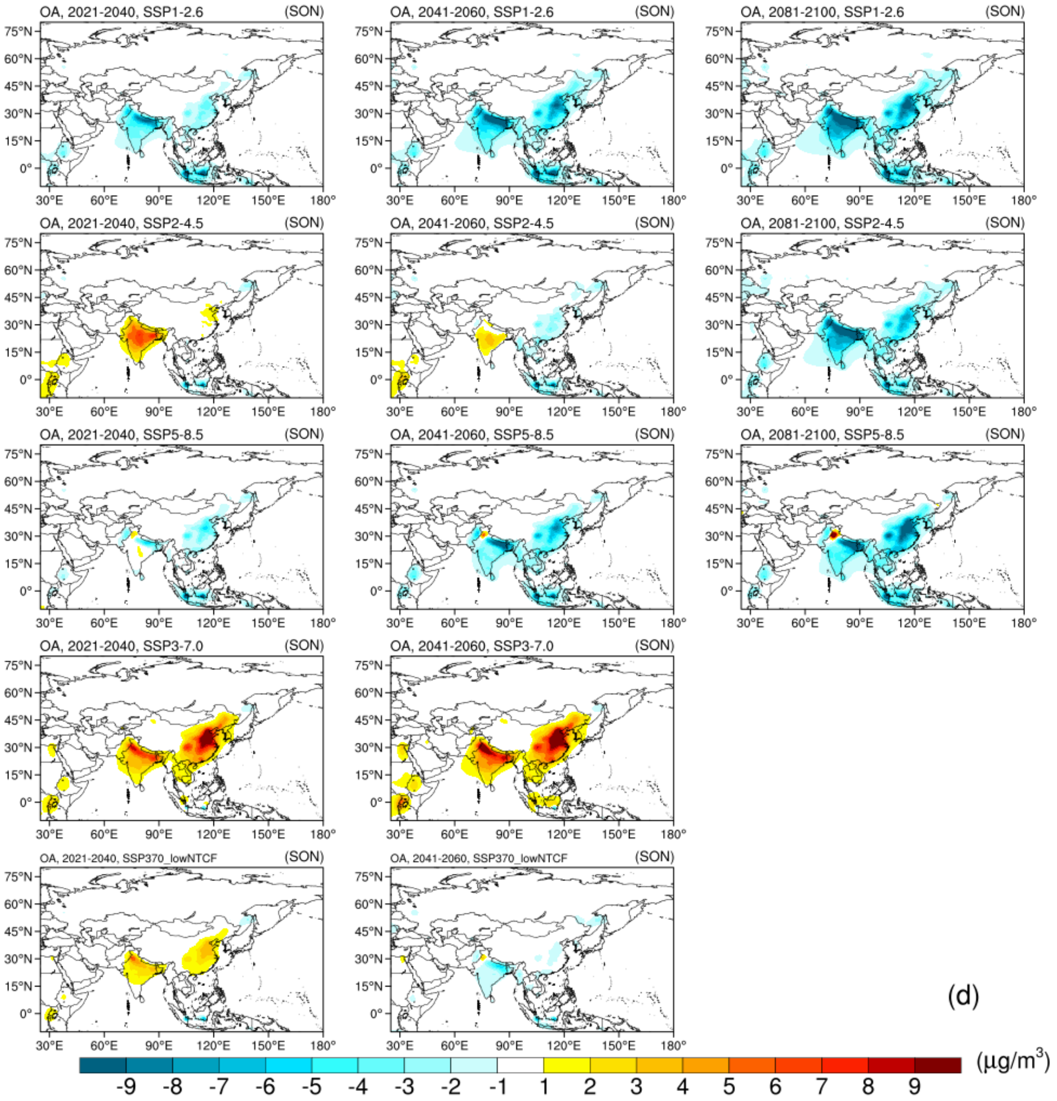
OA, 2021-2040, SSP1-2.6 (SON)
OA, 2041-2060, SSP1-2.6 (SON)
OA, 2081-2100, SSP1-2.6 (SON)
OA, 2021-2040, SSP2-4.5 (SON)
OA, 2041-2060, SSP2-4.5 (SON)
OA, 2081-2100, SSP2-4.5 (SON)
OA, 2021-2040, SSP5-8.5 (SON)
OA, 2041-2060, SSP5-8.5 (SON)
OA, 2081-2100, SSP5-8.5 (SON)
OA, 2021-2040, SSP3-7.0 (SON)
OA, 2041-2060, SSP3-7.0 (SON)
OA, 2021-2040, SSP370_lowNTCF (SON)
OA, 2041-2060, SSP370_lowNTCF (SON)
75°N
60°N
45°N
30°N
15°N
0°
30°E
60°E
90°E
120°E
150°E
180°
(d)
-9 -8 -7 -6 -5 -4 -3 -2 -1 1 2 3 4 5 6 7 8 9
(μg/m3)

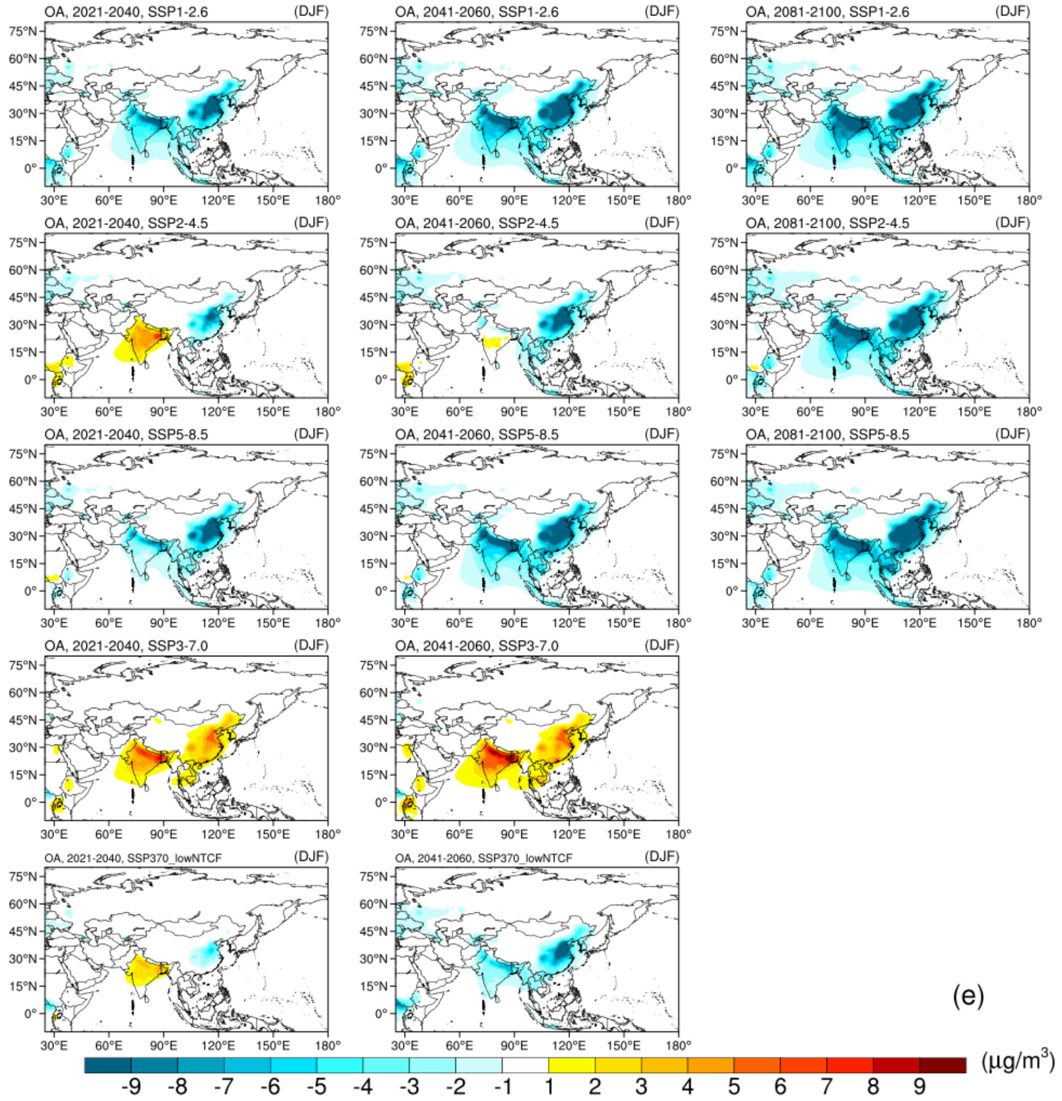

Figure S13 Spatial distribution of projected changes in surface SO_4 concentrations in annual (a), spring (b), summer (c), autumn (d) and winter (e) (units: $\mu g/m^3$) during 2021–2040, 2041–2060 and 2081–2100 under SSPs, relative to 1995–2014.

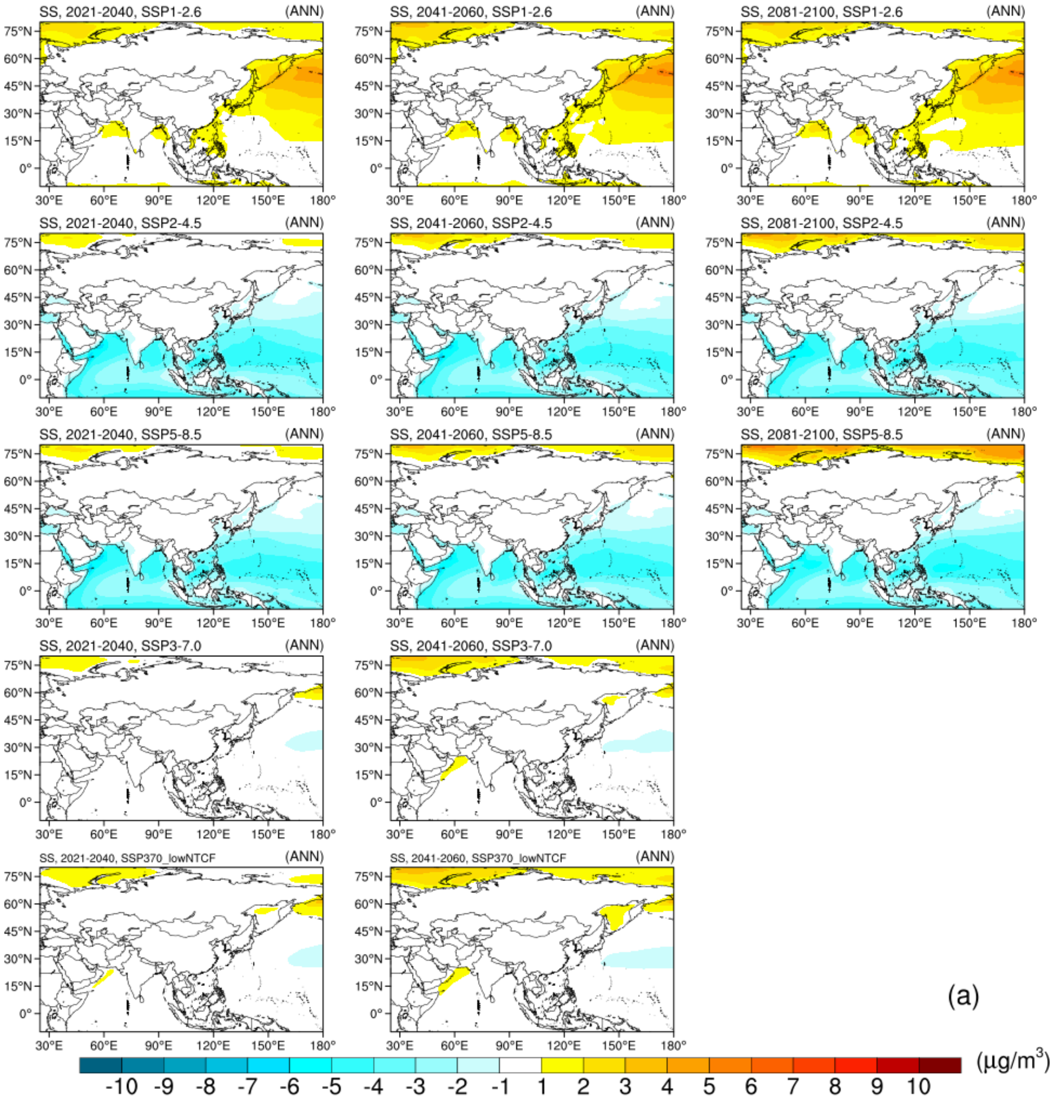

SS, 2021-2040, SSP1-2.6 (ANN)
SS, 2041-2060, SSP1-2.6 (ANN)
SS, 2081-2100, SSP1-2.6 (ANN)
SS, 2021-2040, SSP2-4.5 (ANN)
SS, 2041-2060, SSP2-4.5 (ANN)
SS, 2081-2100, SSP2-4.5 (ANN)
SS, 2021-2040, SSP5-8.5 (ANN)
SS, 2041-2060, SSP5-8.5 (ANN)
SS, 2081-2100, SSP5-8.5 (ANN)
SS, 2021-2040, SSP3-7.0 (ANN)
SS, 2041-2060, SSP3-7.0 (ANN)
SS, 2021-2040, SSP370_lowNTCF (ANN)
SS, 2041-2060, SSP370_lowNTCF (ANN)
75°N 60°N 45°N 30°N 15°N 0°
30°E 60°E 90°E 120°E 150°E 180°
(a)
-10 -9 -8 -7 -6 -5 -4 -3 -2 -1 1 2 3 4 5 6 7 8 9 10
(μg/m3)

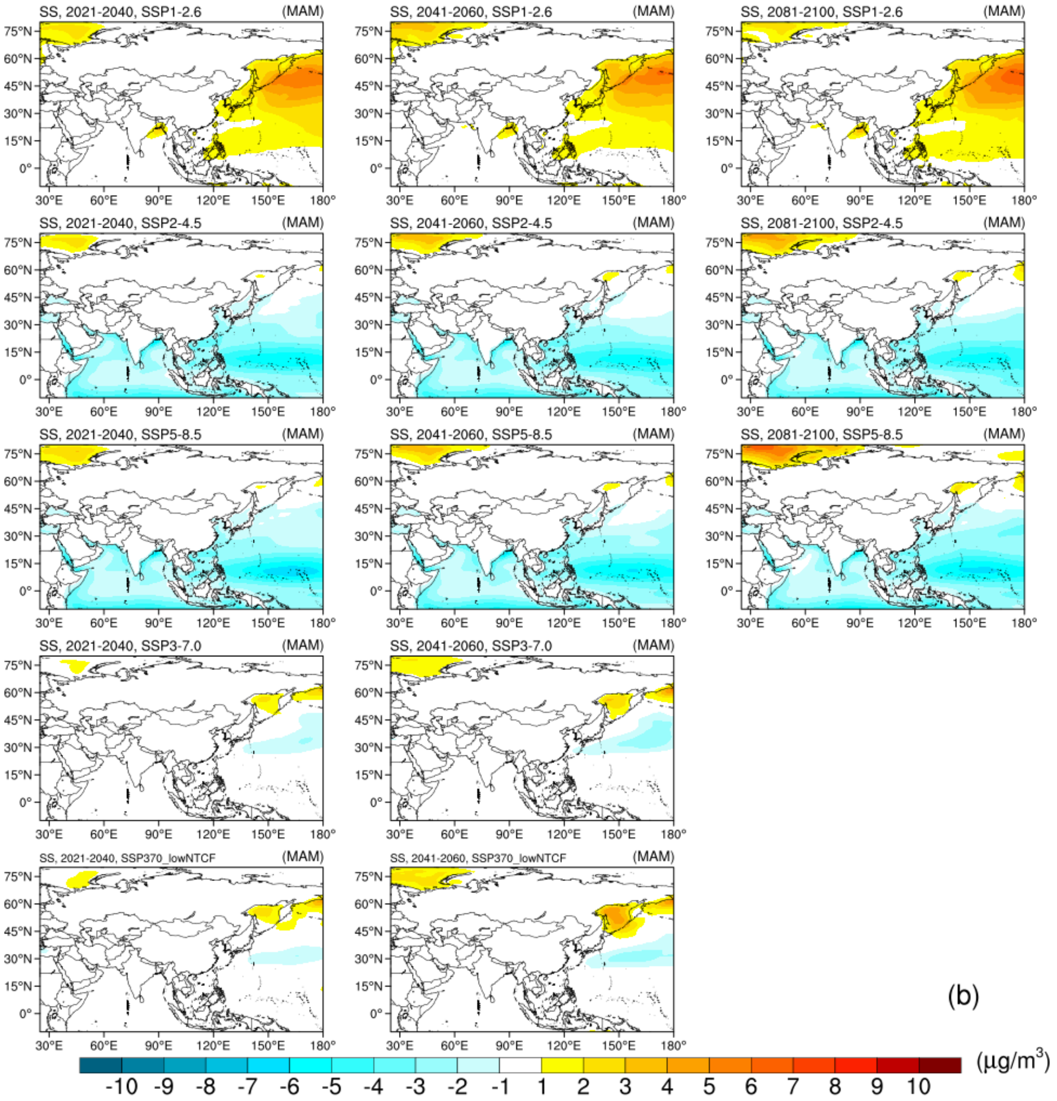
SS, 2021-2040, SSP1-2.6 (MAM)
SS, 2041-2060, SSP1-2.6 (MAM)
SS, 2081-2100, SSP1-2.6 (MAM)
SS, 2021-2040, SSP2-4.5 (MAM)
SS, 2041-2060, SSP2-4.5 (MAM)
SS, 2081-2100, SSP2-4.5 (MAM)
SS, 2021-2040, SSP5-8.5 (MAM)
SS, 2041-2060, SSP5-8.5 (MAM)
SS, 2081-2100, SSP5-8.5 (MAM)
SS, 2021-2040, SSP3-7.0 (MAM)
SS, 2041-2060, SSP3-7.0 (MAM)
SS, 2021-2040, SSP370_lowNTCF (MAM)
SS, 2041-2060, SSP370_lowNTCF (MAM)
75°N 60°N 45°N 30°N 15°N 0°
30°E 60°E 90°E 120°E 150°E 180°
(b)
-10 -9 -8 -7 -6 -5 -4 -3 -2 -1 1 2 3 4 5 6 7 8 9 10
(μg/m3)

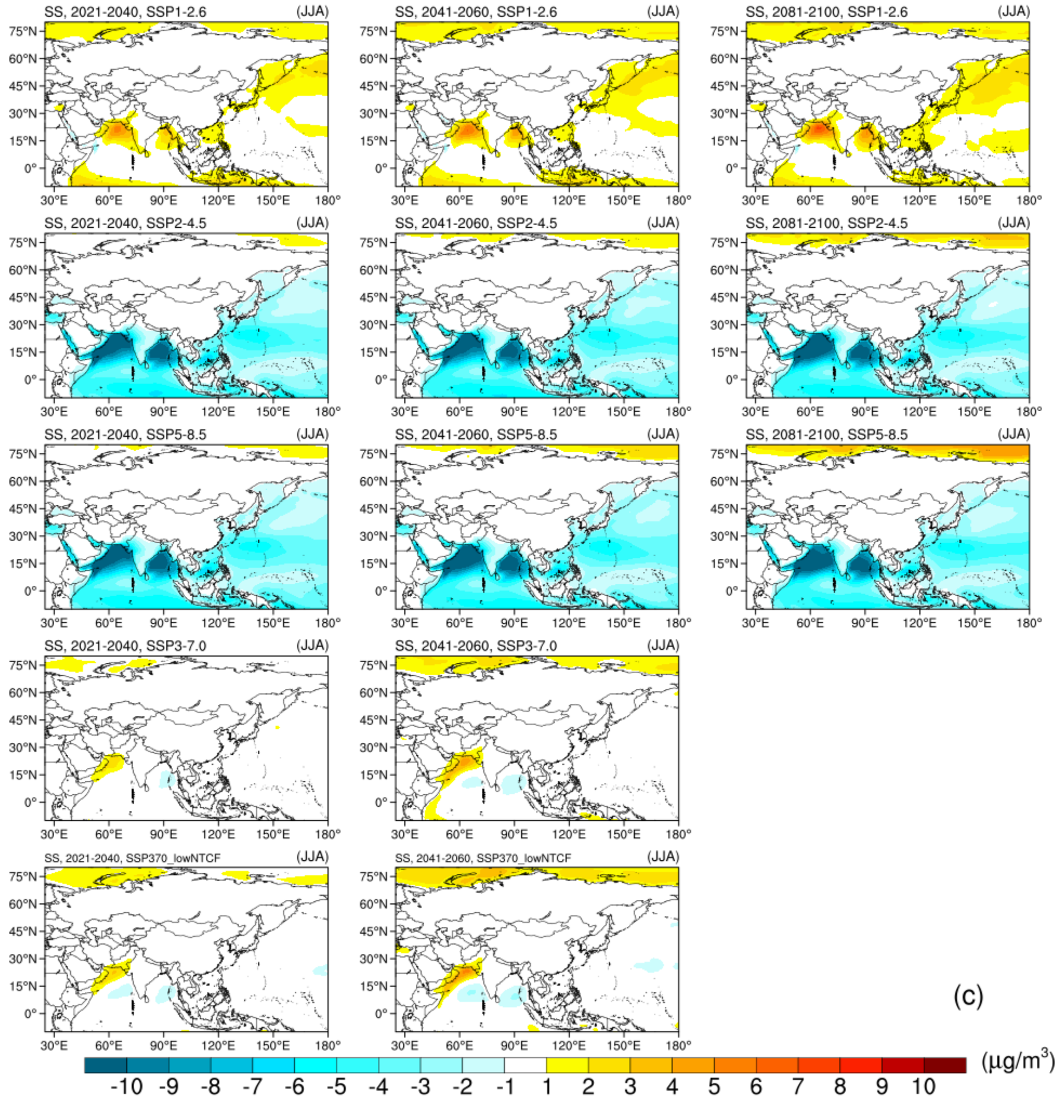
SS, 2021-2040, SSP1-2.6 (JJA)
SS, 2041-2060, SSP1-2.6 (JJA)
SS, 2081-2100, SSP1-2.6 (JJA)
SS, 2021-2040, SSP2-4.5 (JJA)
SS, 2041-2060, SSP2-4.5 (JJA)
SS, 2081-2100, SSP2-4.5 (JJA)
SS, 2021-2040, SSP5-8.5 (JJA)
SS, 2041-2060, SSP5-8.5 (JJA)
SS, 2081-2100, SSP5-8.5 (JJA)
SS, 2021-2040, SSP3-7.0 (JJA)
SS, 2041-2060, SSP3-7.0 (JJA)
SS, 2021-2040, SSP370_lowNTCF (JJA)
SS, 2041-2060, SSP370_lowNTCF (JJA)
75°N 60°N 45°N 30°N 15°N 0°
30°E 60°E 90°E 120°E 150°E 180°
(c)
-10 -9 -8 -7 -6 -5 -4 -3 -2 -1 1 2 3 4 5 6 7 8 9 10
(μg/m3)

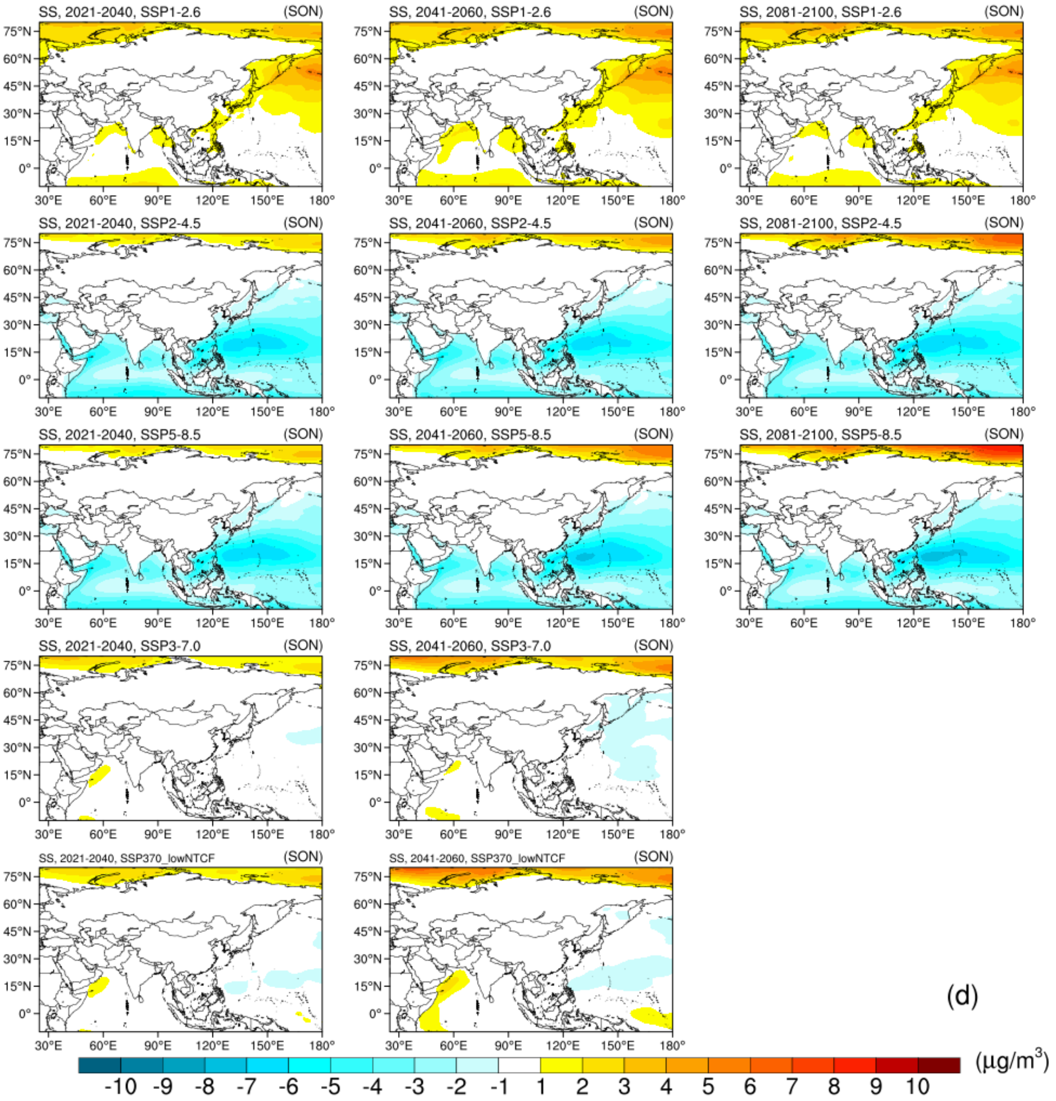

SS, 2021-2040, SSP1-2.6
(SON)
SS, 2041-2060, SSP1-2.6
(SON)
SS, 2081-2100, SSP1-2.6
(SON)
SS, 2021-2040, SSP2-4.5
(SON)
SS, 2041-2060, SSP2-4.5
(SON)
SS, 2081-2100, SSP2-4.5
(SON)
SS, 2021-2040, SSP5-8.5
(SON)
SS, 2041-2060, SSP5-8.5
(SON)
SS, 2081-2100, SSP5-8.5
(SON)
SS, 2021-2040, SSP3-7.0
(SON)
SS, 2041-2060, SSP3-7.0
(SON)
SS, 2021-2040, SSP370_lowNTCF
(SON)
SS, 2041-2060, SSP370_lowNTCF
(SON)
75°N
60°N
45°N
30°N
15°N
0°
30°E
60°E
90°E
120°E
150°E
180°
(d)
-10 -9 -8 -7 -6 -5 -4 -3 -2 -1 1 2 3 4 5 6 7 8 9 10
(μg/m3)

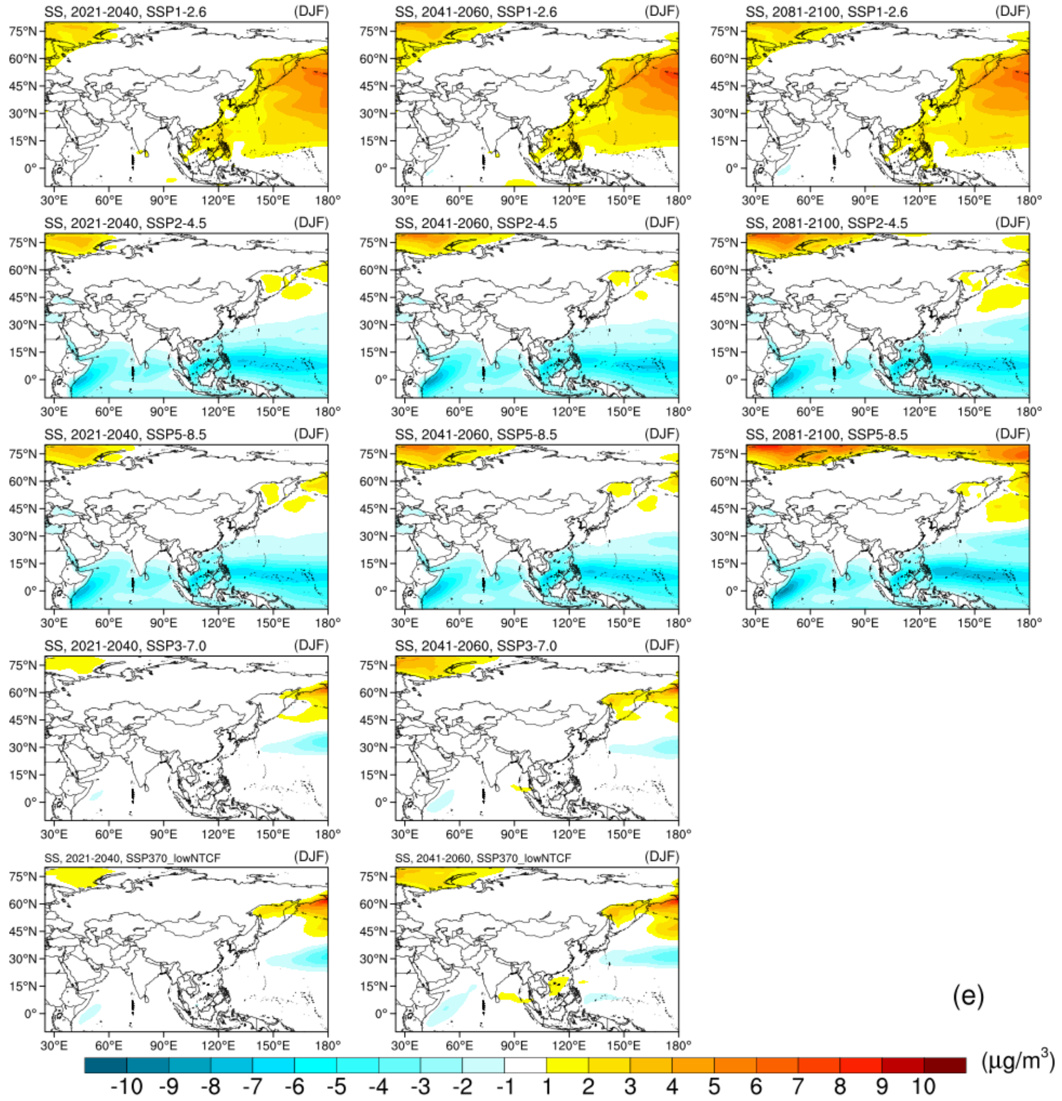

Figure S14 Spatial distribution of projected changes in surface SS concentrations in annual (a), spring (b), summer (c), autumn (d) and winter (e) (units: $\mu g/m^3$) during 2021–2040, 2041–2060 and 2081–2100 under SSPs, relative to 1995–2014.
